# Supplementary material for: PP2A phosphatase regulates Hippo signalling in dual manner
Source: EMBO J. 2026 Jul 7;45(16):5943–71. doi: 10.1038/s44318-026-00850-9 (PMC13482807; doi:10.1038/s44318-026-00850-9)
Supplement: Supplementary file 1 [file 44318_2026_850_MOESM10_ESM.zip › Figure EV2/EV2A_scans.pdf]

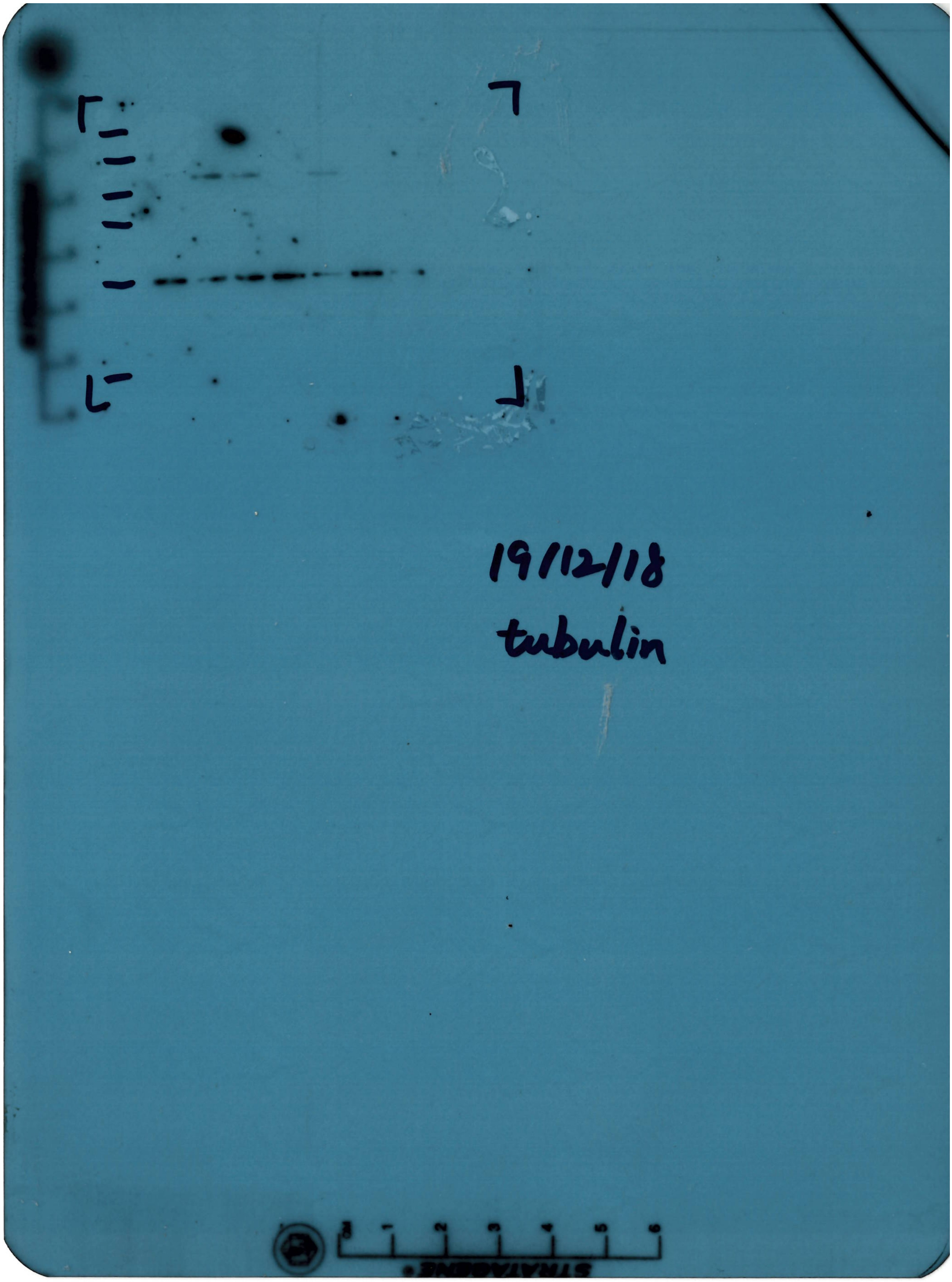
19/12/18
tubulin

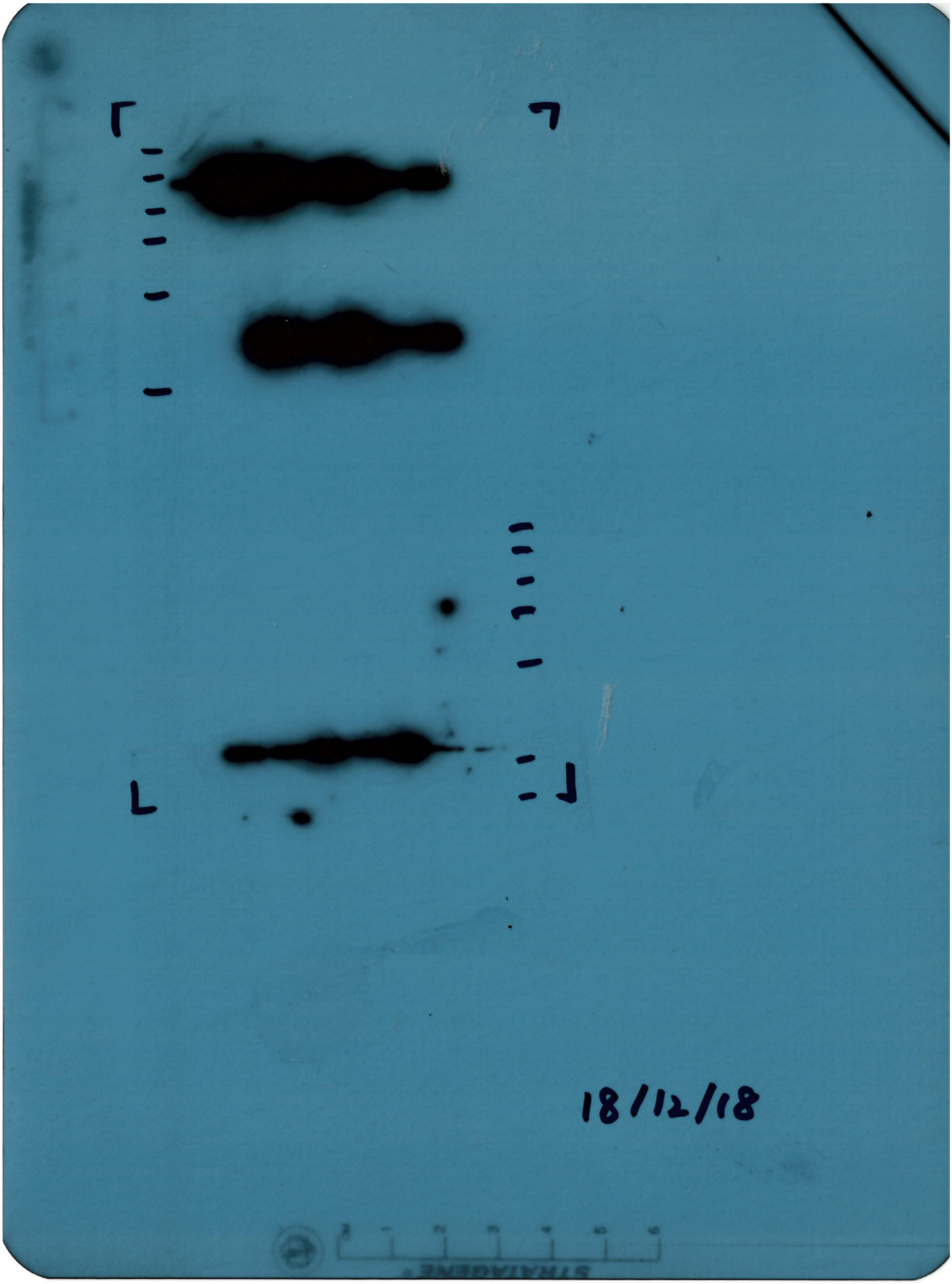
18/12/18

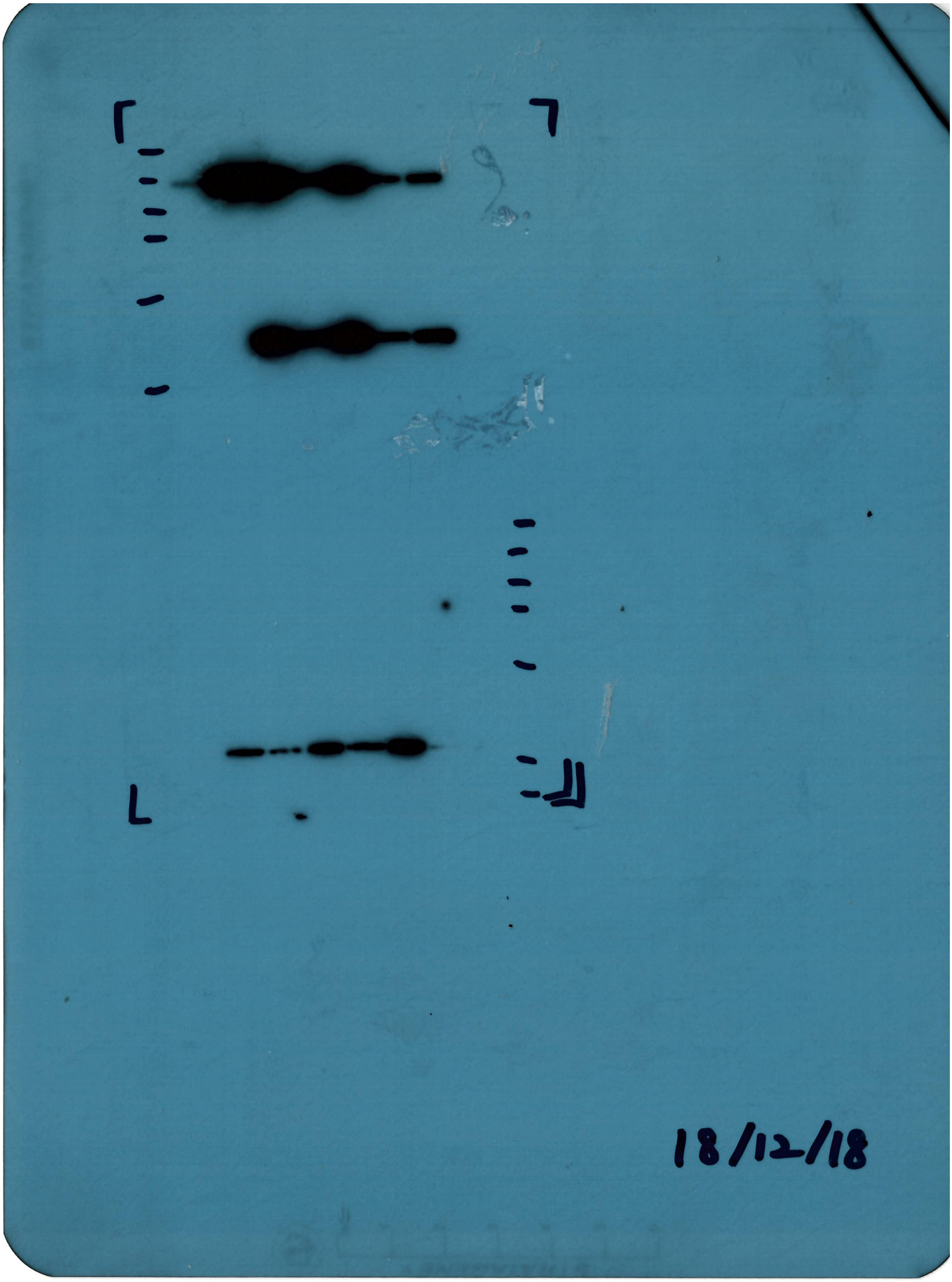
18/12/18

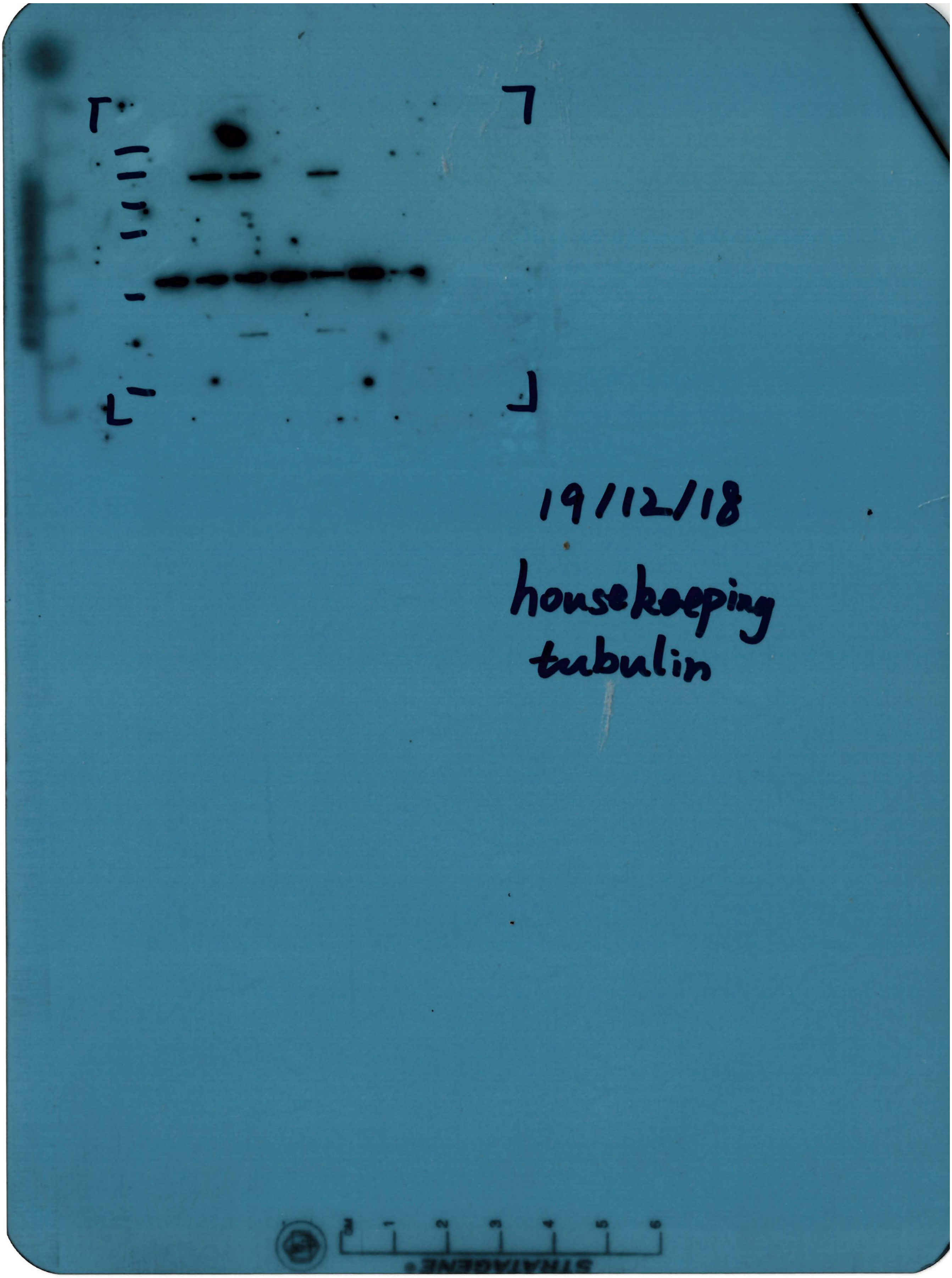
19/12/18
housekeeping
tubulin
STRATAGENE

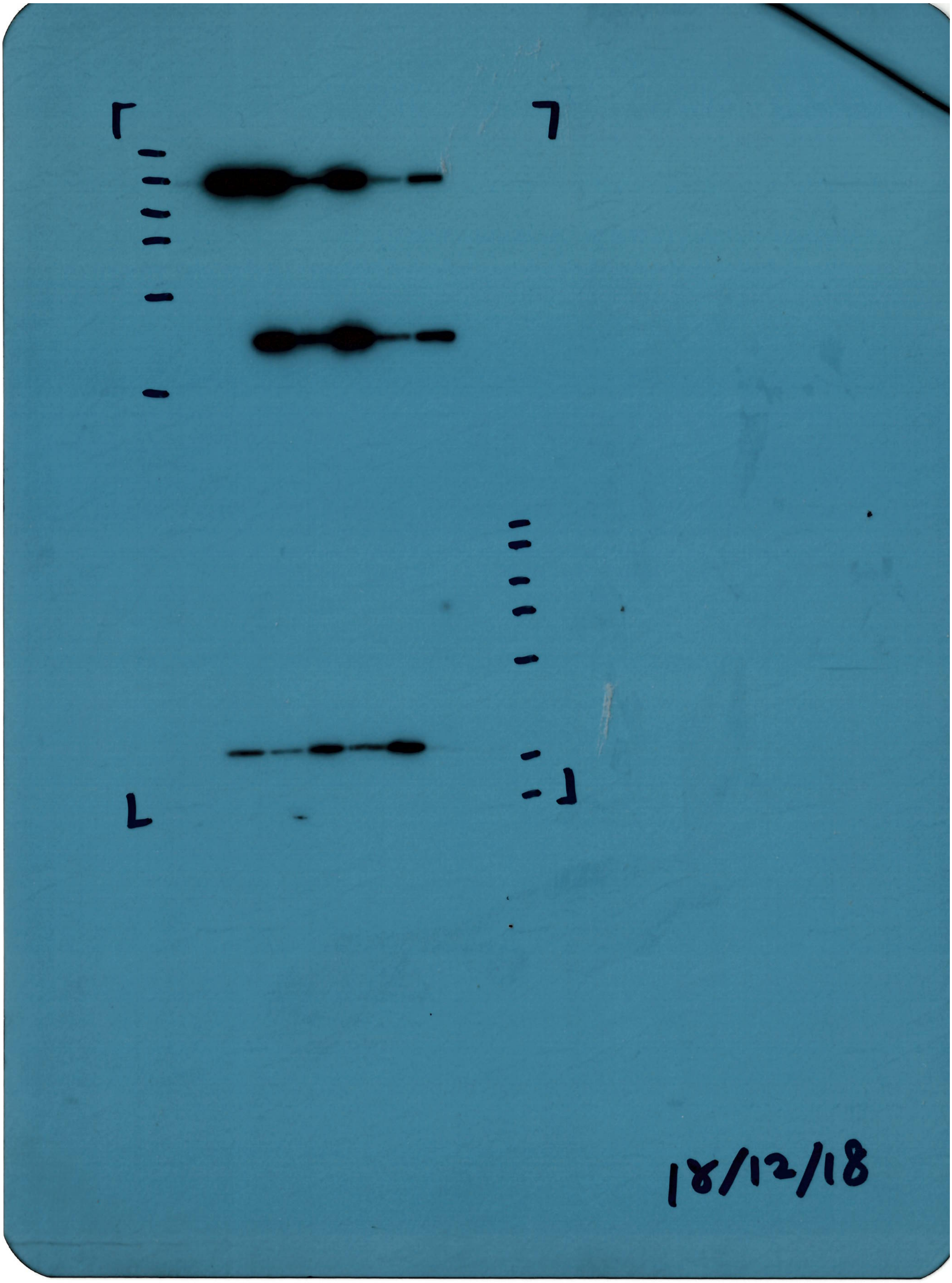
18/12/18
