# Supplementary material for: PP2A phosphatase regulates Hippo signalling in dual manner
Source: EMBO J. 2026 Jul 7;45(16):5943–71. doi: 10.1038/s44318-026-00850-9 (PMC13482807; doi:10.1038/s44318-026-00850-9)

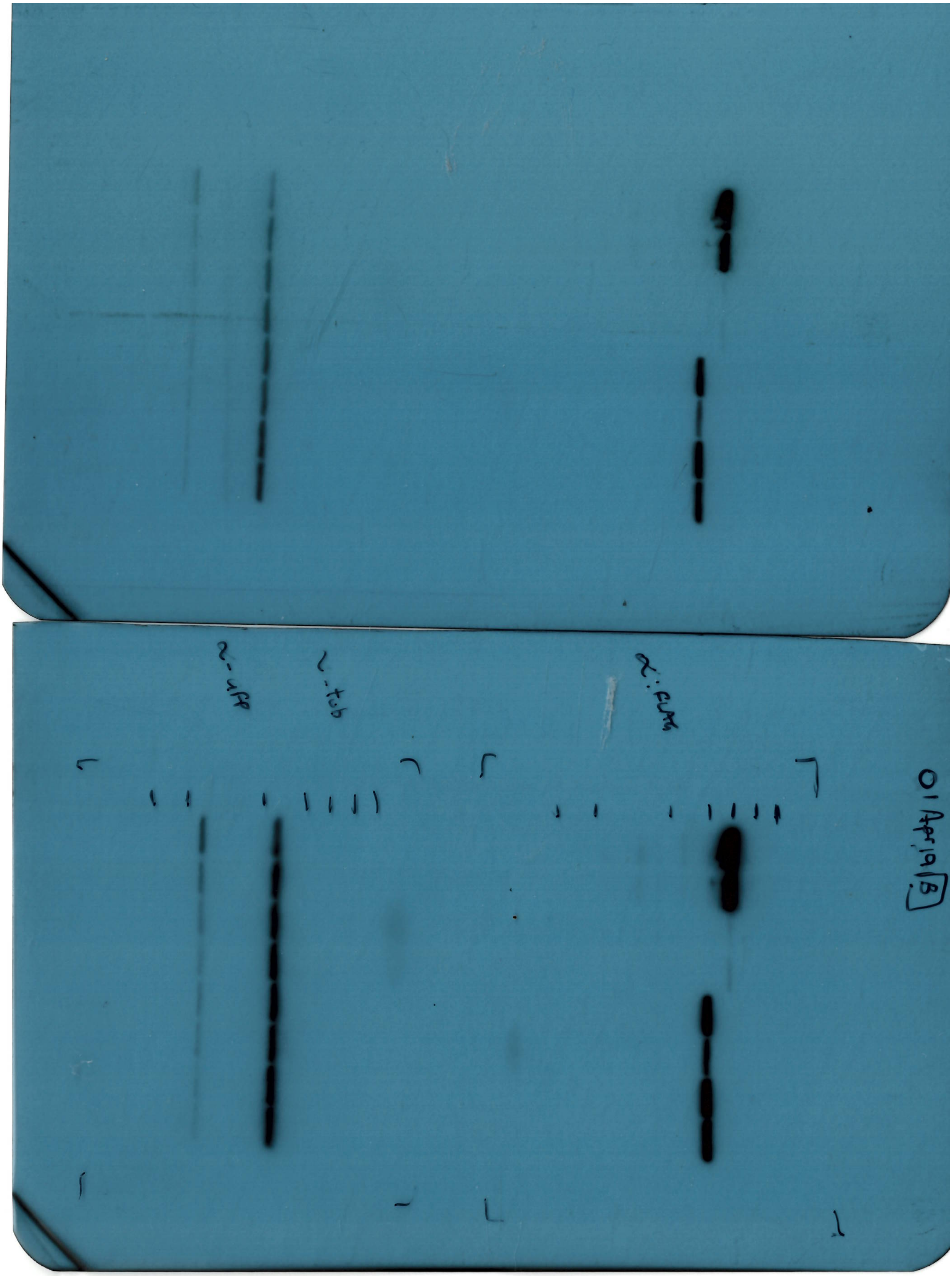
01 Apr 19

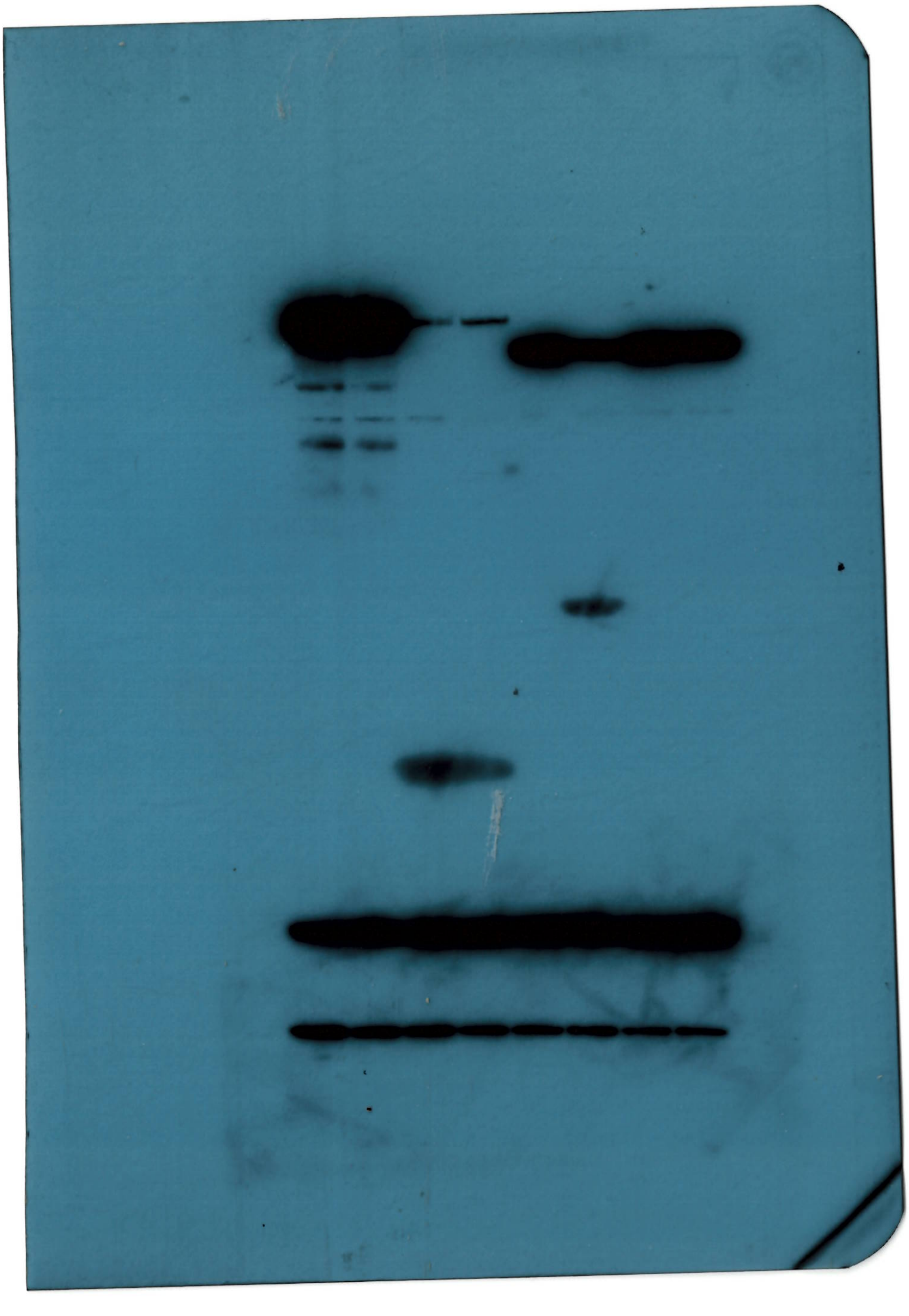

Supplement: Supplementary file 4 [file 44318_2026_850_MOESM13_ESM.zip › Figure EV5/EV5A_scans.pdf]
