# Supplementary material for: PP2A phosphatase regulates Hippo signalling in dual manner
Source: EMBO J. 2026 Jul 7;45(16):5943–71. doi: 10.1038/s44318-026-00850-9 (PMC13482807; doi:10.1038/s44318-026-00850-9)

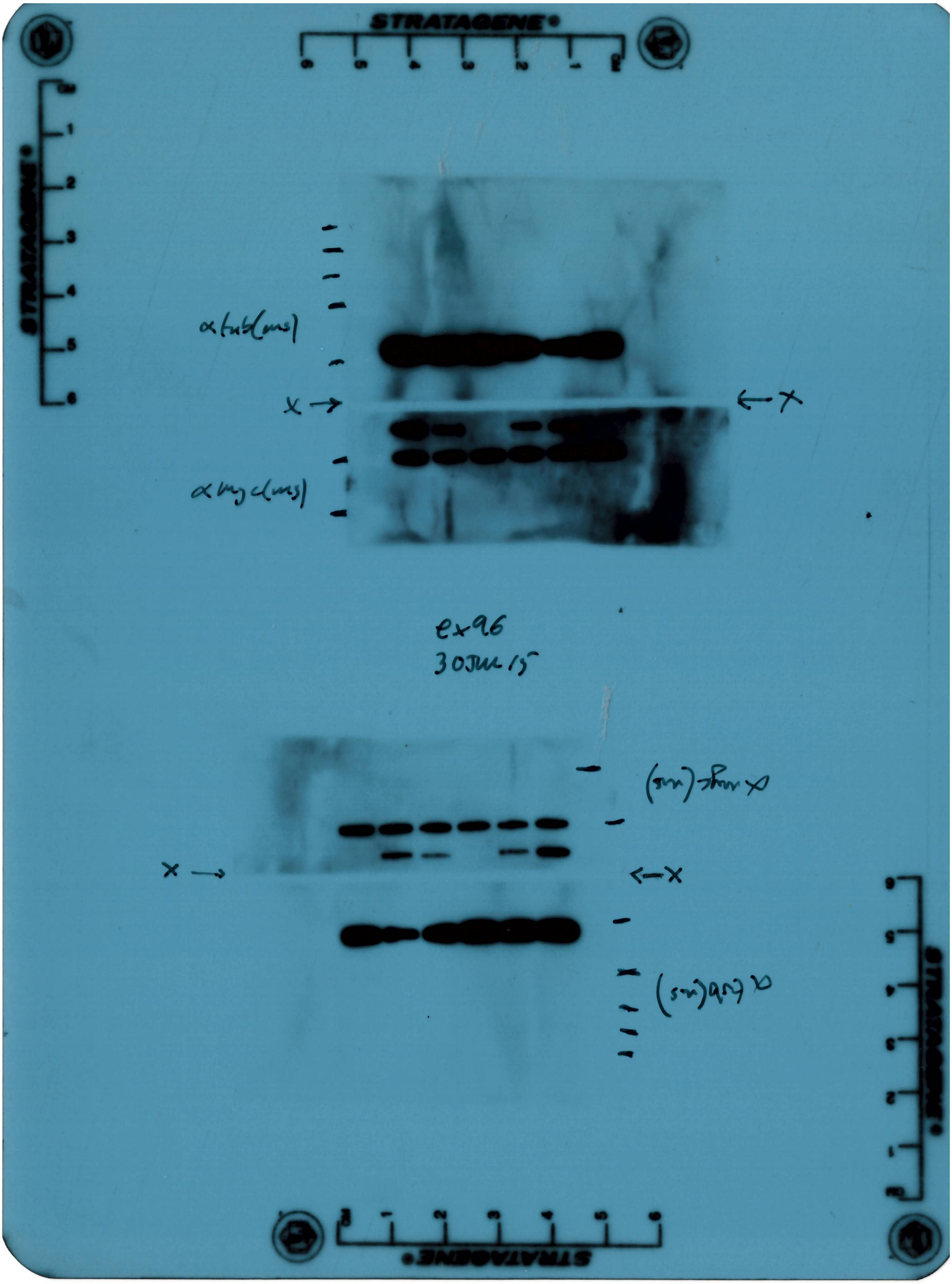
STRATAGENE
α tub (ms)
α myc (ms)
ex96
30Jun15

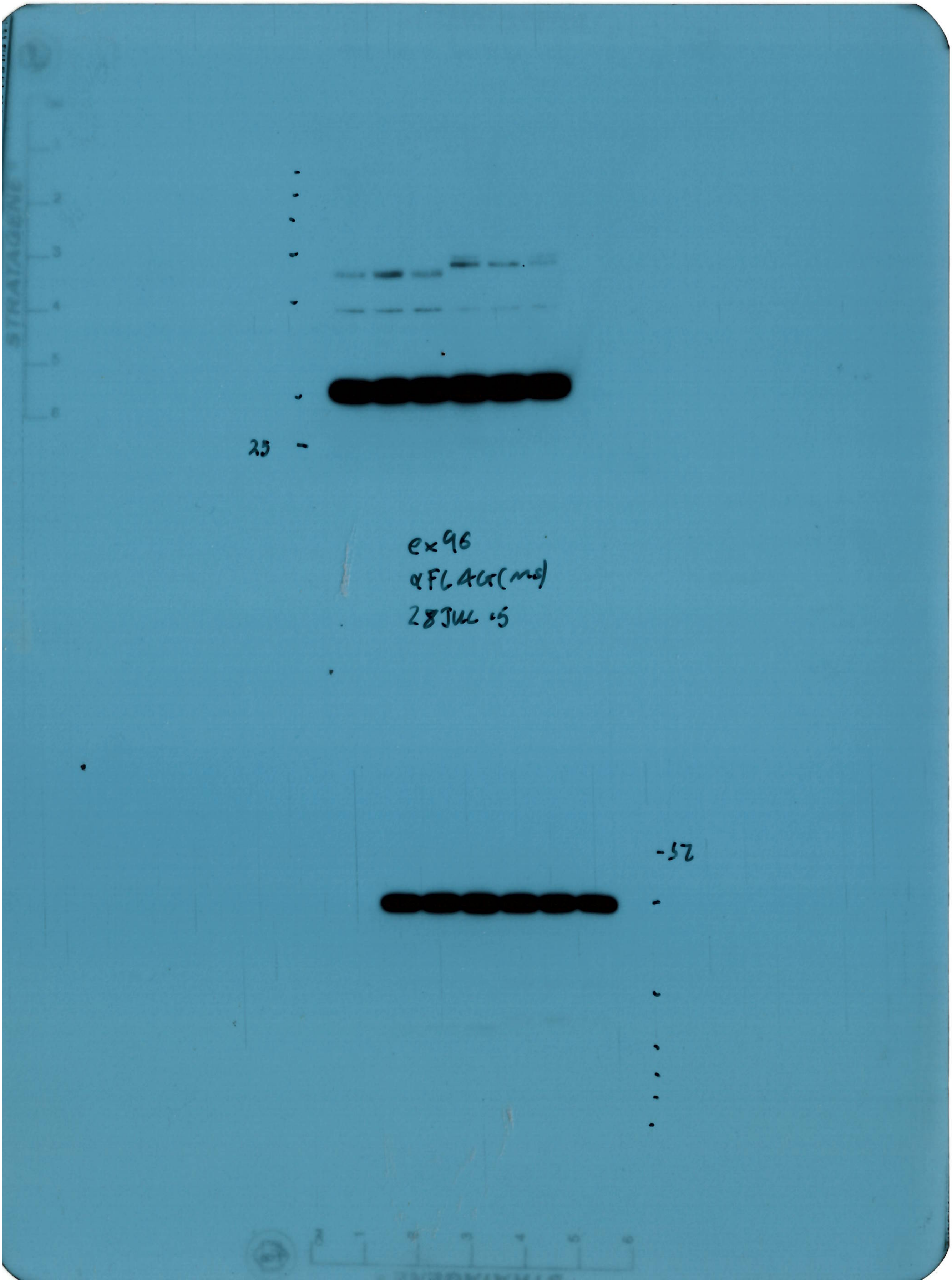
25
ex96
αFLAG(ms)
28JUL 05
25

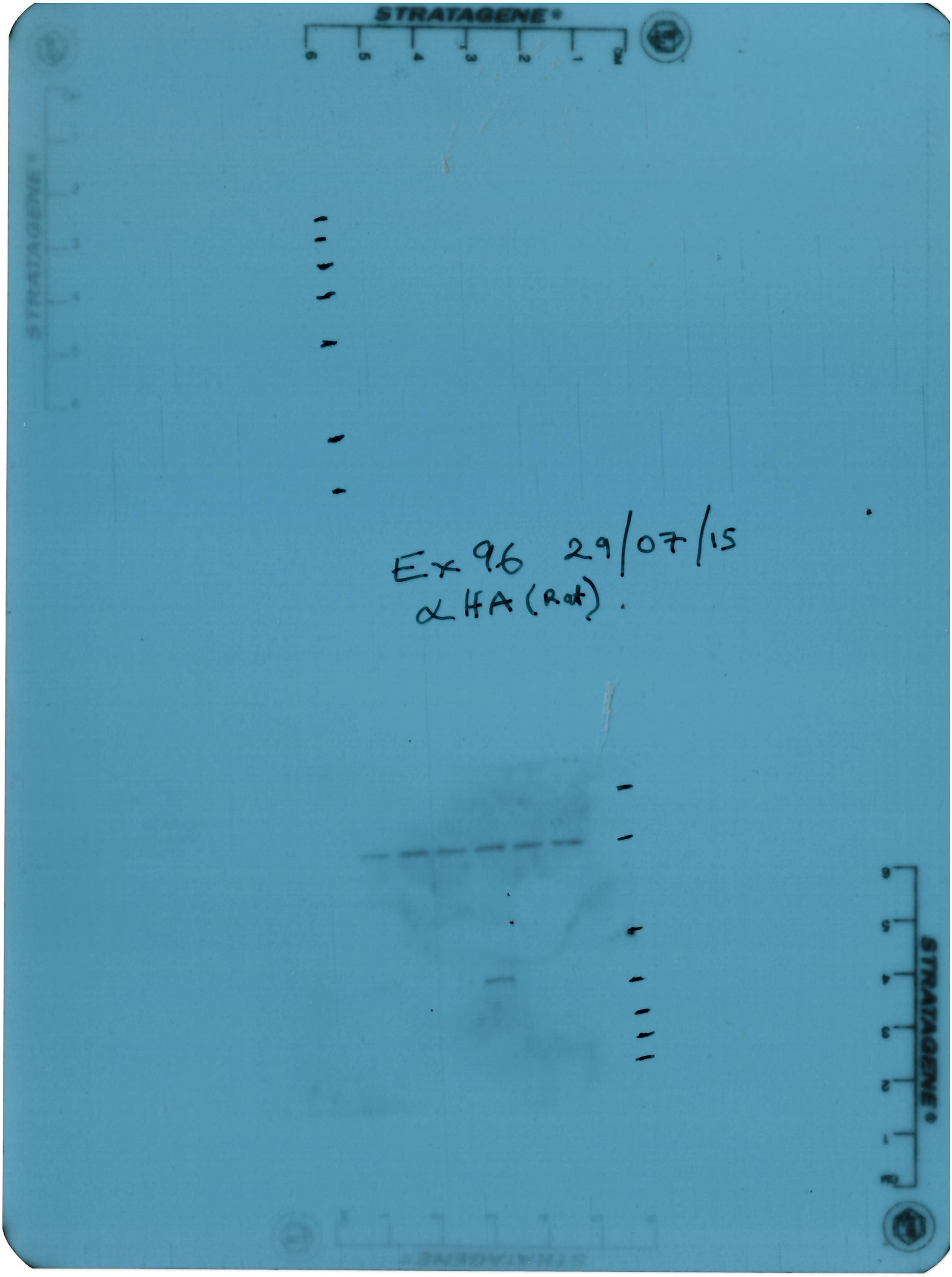
STRATAGENE®
Ex 96 29/07/15
αHA (Rat).

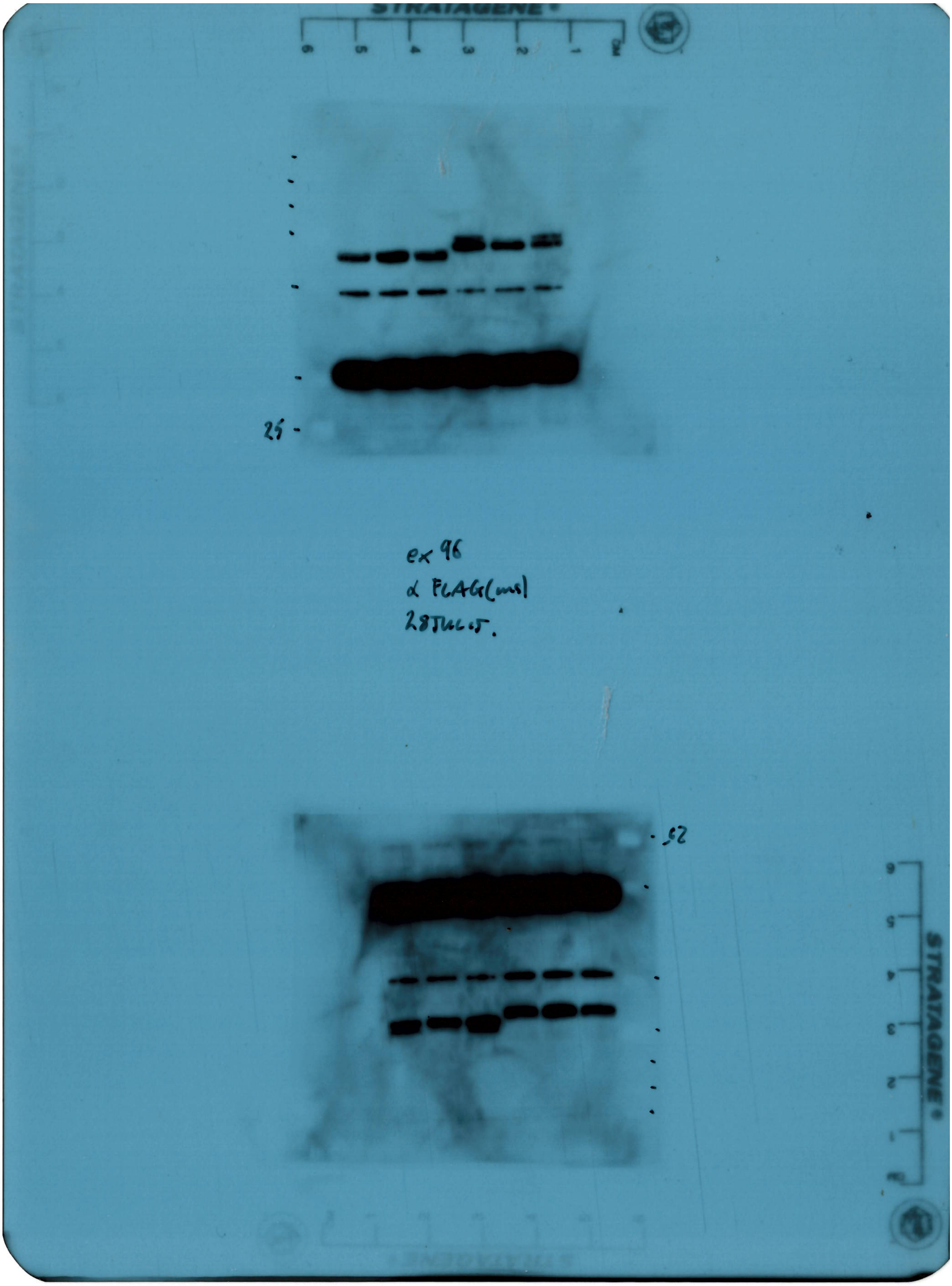
STRATAGENE
25
ex 96
α FLAG(ms)
28JUL05.
62

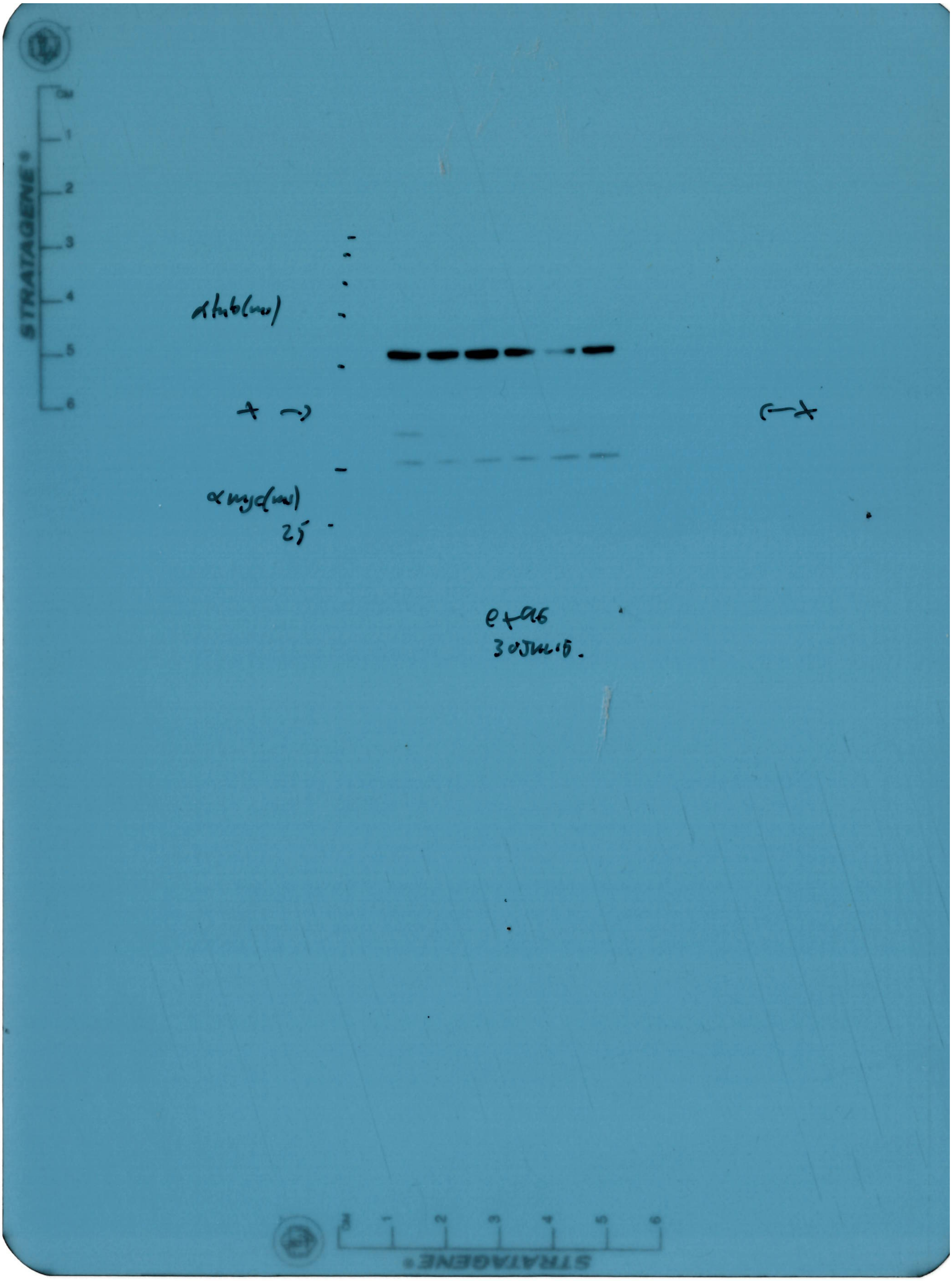

STRATAGENE®
αtub(ms)
αmyc(ms)
25
e+a6
30Jun15.
STRATAGENE®

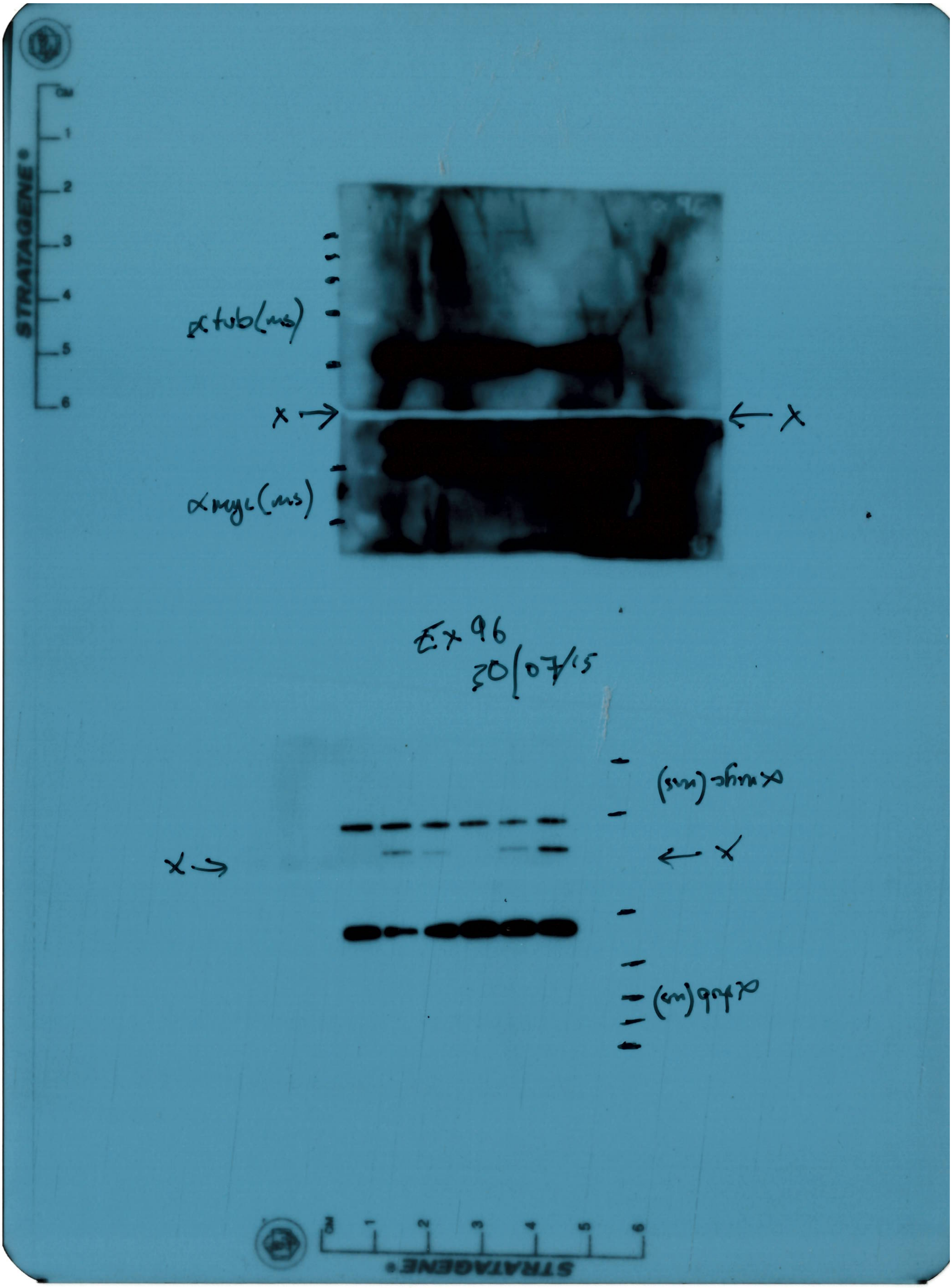
STRATAGENE
αtub(ms)
X →
← X
αmyc(ms)
Ex 96
30/07/15

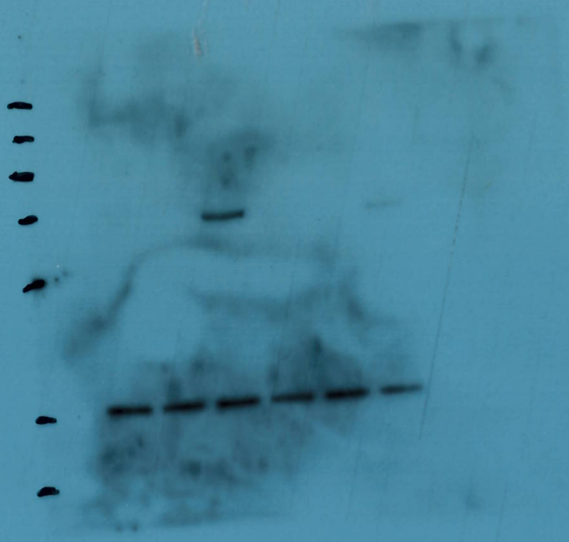

Ex 96 29/07/15

αHA (Rat)

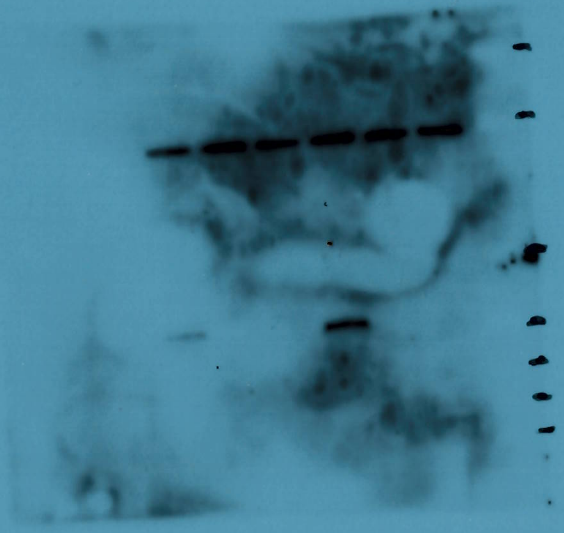

Supplement: Supplementary file 7 [file 44318_2026_850_MOESM4_ESM.zip › Figure 2/2N_Scans.pdf]
