# Supplementary material for: PP2A phosphatase regulates Hippo signalling in dual manner
Source: EMBO J. 2026 Jul 7;45(16):5943–71. doi: 10.1038/s44318-026-00850-9 (PMC13482807; doi:10.1038/s44318-026-00850-9)
Supplement: Supplementary file 1 [file 44318_2026_850_MOESM15_ESM.pdf]

Expanded View Figures

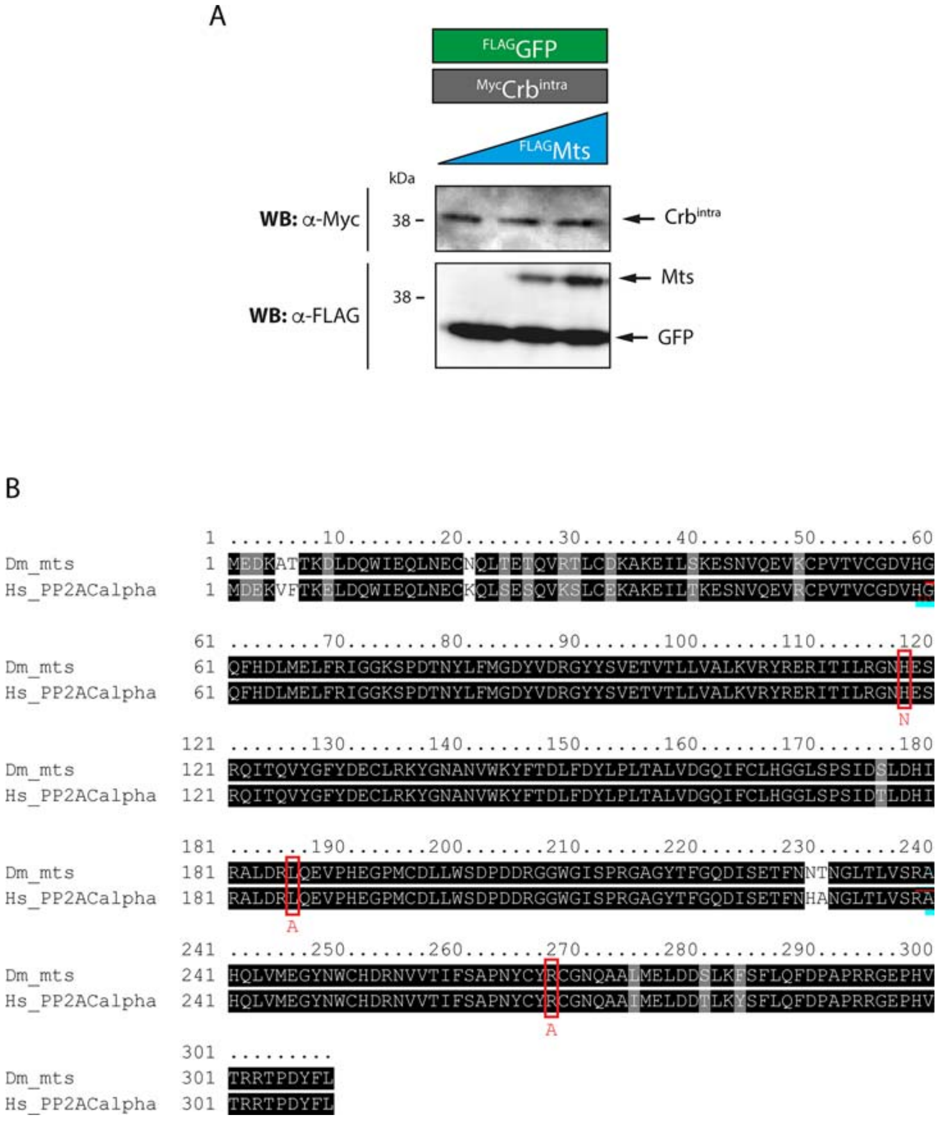

Figure EV1. PP2A does not regulate Crb protein levels and is highly conserved with human PP2Acα.

(**A**) S2 cells were transfected with the indicated constructs and immunoblotted with the indicated antibodies 48 h after transfection. Crb protein levels are unaffected by co-expression with increasing levels of Mts. (**B**) Protein sequence alignment of Mts (Dm_mts) and human PP2ACα (Hs_PP2ACalpha) with mutations used in this study marked in red. Source data are available online for this figure.

© The Author(s)

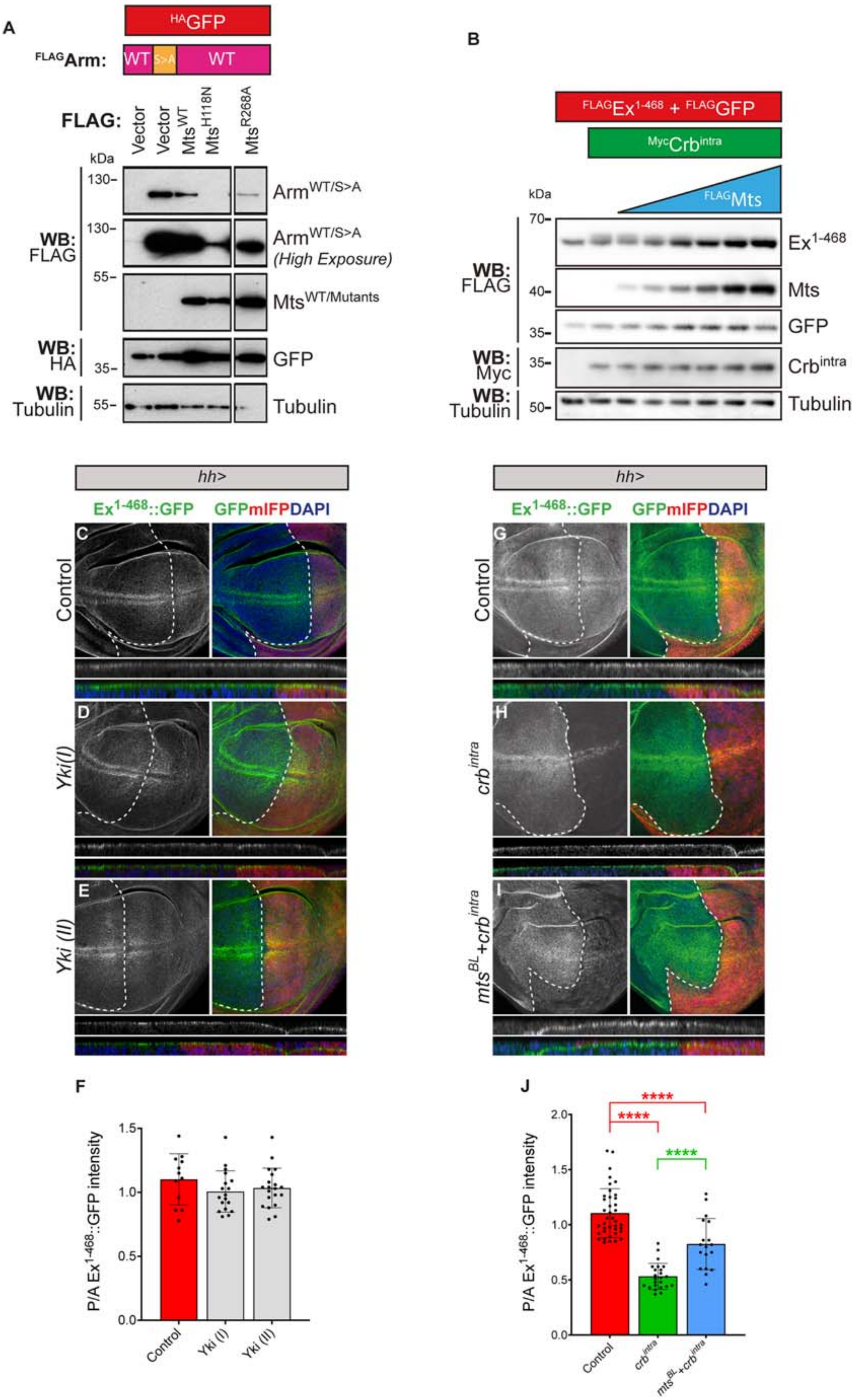
A
HAGFP
FLAGArm:
WT
S>A
WT
FLAG:
Vector
Vector
MtsWT
MtsH118N
MtsR268A
kDa
130
130
55
35
55
WB: FLAG
WB: HA
WB: Tubulin
ArmWT/S>A
ArmWT/S>A (High Exposure)
MtsWT/Mutants
GFP
Tubulin
B
FLAGEx1-468 + FLAGGFP
MycCrbintra
FLAGMts
kDa
70
40
35
35
50
WB: FLAG
WB: Myc
WB: Tubulin
Ex1-468
Mts
GFP
Crbintra
Tubulin
hh>
Ex1-468::GFP
GFPmIFPDAPI
C
Control
D
Yki(I)
E
Yki (II)
G
Control
H
crbintra
I
mtsBL+crbintra
F
P/A Ex1-468::GFP intensity
1.5
1.0
0.5
0.0
Control
Yki (I)
Yki (II)
J
****
****
****
2.0
1.5
1.0
0.5
0.0
Control
crbintra
mtsBL+crbintra

Figure EV2. PP2A-mediated regulation of Arm and of Ex in the presence of Crb^{intra}.

(**A**) Assessment of PP2A catalytic activity in vitro. S2 cells were transfected with the indicated constructs 48 h prior to cell lysis and processing for immunoblot analysis with the indicated antibodies. Note that Arm is constitutively degraded in S2 cells in a phosphorylation-dependent manner. Mts^{WT} and Mts^{R268A} stabilise Arm^{WT}, therefore indicating that Mts^{R268A} is catalytically active. Mts^{H118N} is unable to stabilise Arm^{WT}, demonstrating that Mts^{H118N} is catalytically inactive. $Arm^{S>A}$ contains a mutation in the Arm phosphorylation site, rendering it refractory to protein degradation. (**B**) PP2A stabilises Ex in the presence of Crb^{intra} in a dose-dependent manner. GFP and Tubulin were used as transfection and loading controls, respectively. (**C–E**) In vivo, the Ex reporter does not respond to the transcriptional activity of Yki. (**G–I**) Mts-mediated regulation of Ex in vivo reporter. (**C–E**, **G–I**) Confocal micrographs show XY (top) and transverse sections (bottom) of third instar wing imaginal discs expressing *ubi-*$Ex^{1\text{-}468}$*::GFP* (green) and *UAS-mIFP* (red) alone (control, **C**, **G**) or in combination with indicated transgenes in the posterior compartment, under the control of the *hh-Gal4* driver. Nuclei were stained with DAPI (blue). Dashed white lines depict the AP boundary. Scale bars correspond to 50 µm. (**F**) Quantification of Ex in vivo reporter upon Yki expression. The bar chart depicts the mean ratio of posterior to anterior $Ex^{1\text{-}468}$::GFP intensity for the indicated genotypes, with all data points represented. One-way ANOVA was not statistically significant. (**J**) Quantification of Ex in vivo reporter upon Mts^{BL} expression. Bar charts depict the geometric mean ratio of posterior-to-anterior $Ex^{1\text{-}468}$::GFP intensity for the indicated genotypes, with all data points represented. Significance was assessed by a one-way ANOVA conducted on log-transformed data, followed by Tukey's multiple comparisons post hoc test. $n \geq 17$ for all genotypes. ****$P < 0.0001$. Exact P values for all comparisons are listed in Dataset EV1. Expression of mts^{BL} rescued Ex protein levels from Crb-dependent degradation. Source data are available online for this figure.

© The Author(s)

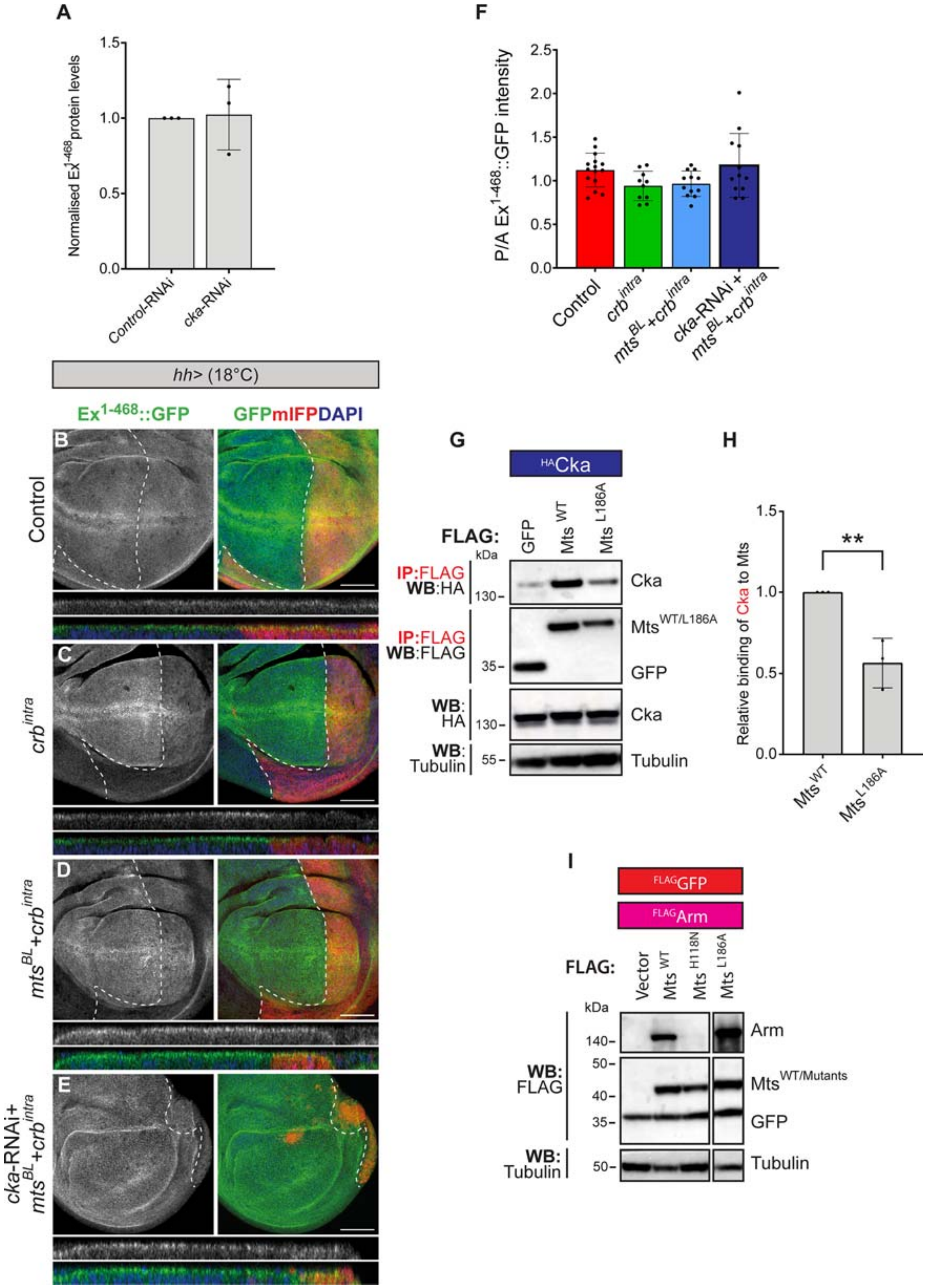
A
Normalised Ex1-468 protein levels
Control-RNAi
cka-RNAi
F
P/A Ex1-468::GFP intensity
Control
crbintra
mtsBL+crbintra
cka-RNAi + mtsBL+crbintra
hh> (18°C)
Ex1-468::GFP
GFPmIFPDAPI
B
Control
C
crbintra
D
mtsBL+crbintra
E
cka-RNAi+ mtsBL+crbintra
G
HACka
FLAG:
GFP
Mts WT
Mts L186A
kDa
IP:FLAG WB:HA
130
Cka
IP:FLAG WB:FLAG
35
MtsWT/L186A
GFP
WB: HA
WB: Tubulin
55
Tubulin
H
Relative binding of Cka to Mts
**
MtsWT
MtsL186A
I
FLAGGFP
FLAGArm
Vector
Mts H118N
140
50
40
Arm
MtsWT/Mutants
WB: FLAG

Figure EV3. Assessing the role of Cka in Crb-dependent regulation of Ex.

(**A**) Quantification of in vitro $Ex^{1\text{-}468}$ protein levels of the representative Western blot depicted in Fig. 1J. The bar chart depicts normalised $Ex^{1\text{-}468}$ levels (to the corresponding Mts^{WT}+Crb^{intra}+*lacZ*-RNAi sample) from three independent experiments. No significant difference was observed between Mts^{WT}+Crb^{intra}+*lacZ*-RNAi and Mts^{WT}+Crb^{intra}+*ckaZ*-RNAi when assessed using an unpaired *t* test. (**B**–**E**) Role of Cka in Ex regulation. Confocal micrographs show XY (top) and transverse sections (bottom) of third instar wing imaginal discs expressing *ubi-Ex*$^{1\text{-}468}$*::GFP* (green) and *UAS-mIFP* (red) alone (control, **A**) or in combination with indicated transgenes in the posterior compartment, under the control of the *hh-Gal4* driver. Crosses were reared at 18 °C. Nuclei were stained with DAPI (blue). Dashed white lines depict the AP boundary. Scale bars correspond to 50 µm. (**F**) Quantification of Ex reporter levels. The bar chart depicts the mean ratio of posterior to anterior $Ex^{1\text{-}468}$::GFP intensity for the indicated genotypes, with all data points included. Significance was assessed by a one-way ANOVA. $n \geq 10$ for all genotypes. (**G**) Cka interaction with PP2A catalytic subunit in vitro. S2 cells were transfected with the indicated constructs. Following lysis, FLAG-tagged proteins were purified using FLAG-agarose beads. Lysates and eluates were processed for western blotting analysis using the indicated antibodies. (**H**) Quantification of Cka:Mts interaction. The bar chart depicts the levels of Cka bound to Mts^{L186A} relative to Mts^{WT} per experiment. Significance was assessed by a paired *t* test. $n = 3$ independent experiments. $^{**}P < 0.01$. Exact *P* values for all comparisons are listed in Dataset EV1. (**I**) Assessment of catalytic activity of STRIPAK-deficient PP2A mutant. S2 cells were transfected with the indicated constructs 48 h prior to cell lysis and processing for immunoblot analysis with the indicated antibodies. Mts^{L186A} stabilises Arm^{WT}, indicating that Mts^{L186A} is catalytically active. Source data are available online for this figure.

© The Author(s)

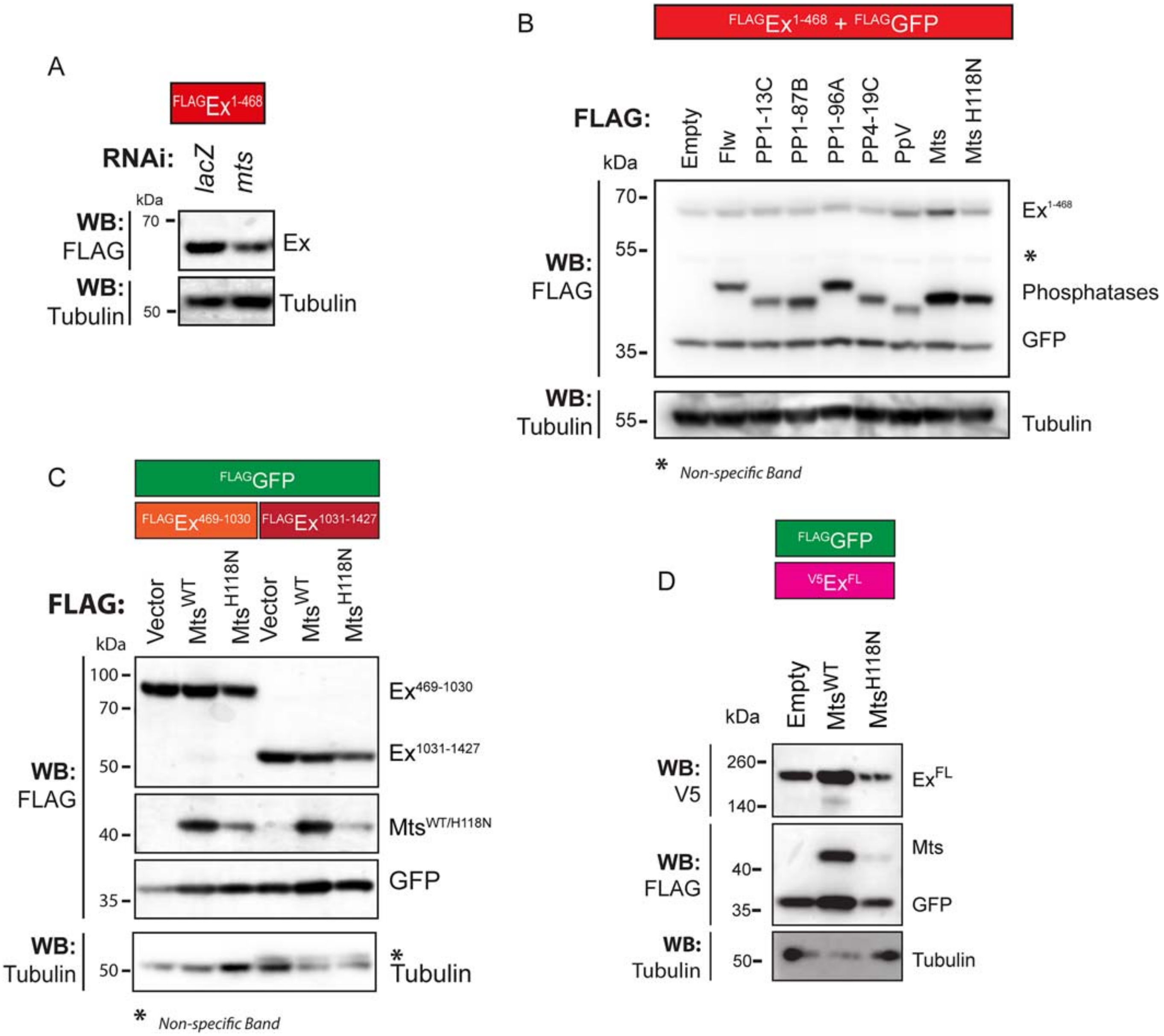

Figure EV4. PP2A regulates Ex proteostasis via its N-terminal region.

If mentioned, S2 cells were treated with the indicated dsRNA for 24 h prior to transfection with the indicated constructs. (**A**) Knocking down *mts* reduces steady-stateEx^{1-468} protein levels. (**B**) In the absence of Crb, Mts was able to increase Ex levels, while none of the catalytic subunits of the other phosphatases influenced steady-state Ex levels. (**C**) Mts^{WT} did not have any effect on $Ex^{469-1030}$ and $Ex^{1031-1427}$ levels. (**D**) Mts^{WT}, but not Mts^{H118N}, was able to increase Ex^{FL} protein levels in conditions where Crb function is dispensable. Non-specific bands are denoted with *. Source data are available online for this figure.

 © The Author(s)

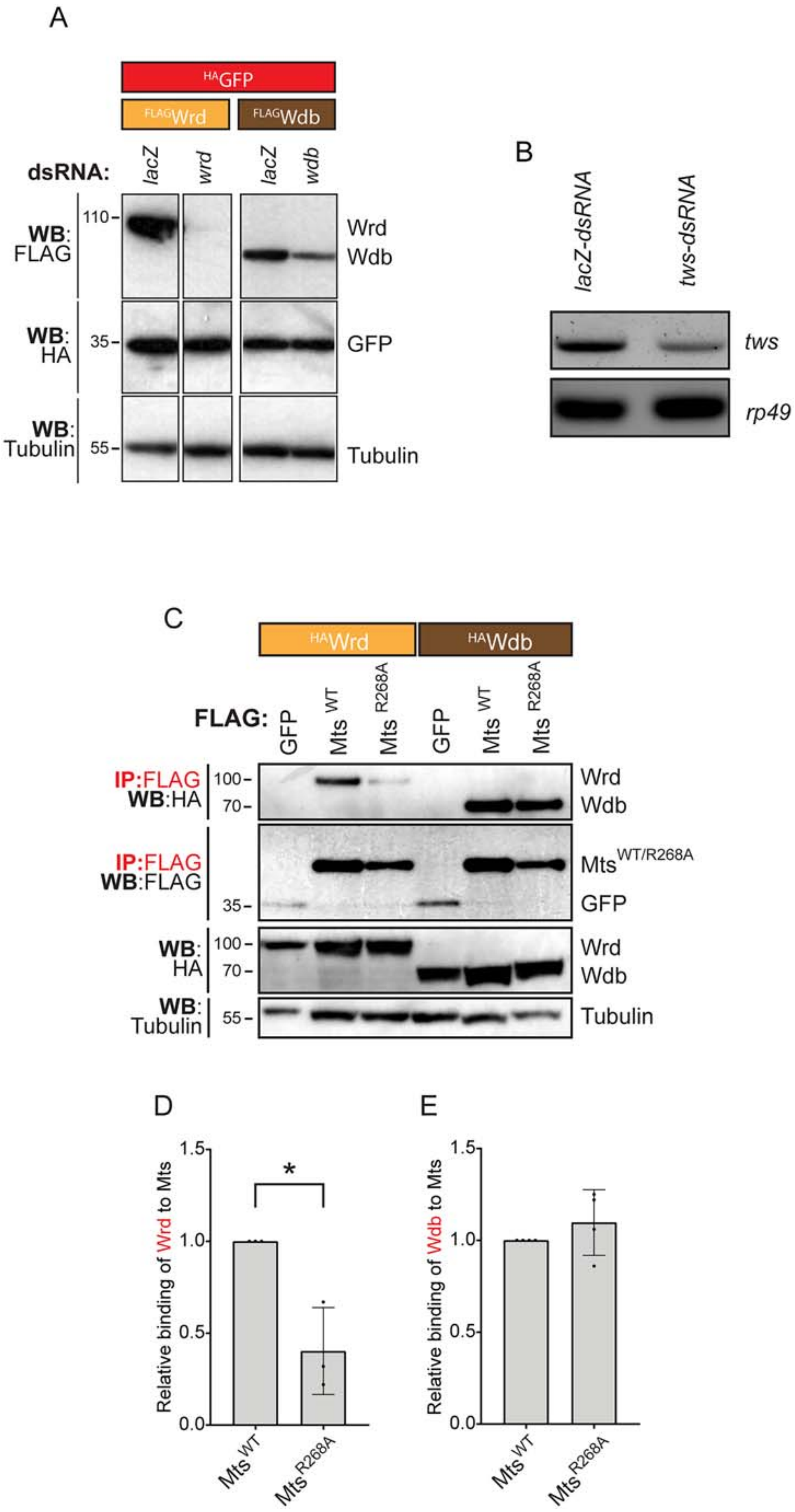
A
HAGFP
FLAGWrd
FLAGWdb
dsRNA:
lacZ
wrd
lacZ
wdb
WB: FLAG
110
Wrd
Wdb
WB: HA
35
GFP
WB: Tubulin
55
Tubulin
B
lacZ-dsRNA
tws-dsRNA
tws
rp49
C
HAWrd
HAWdb
FLAG:
GFP
Mts WT
Mts R268A
GFP
Mts WT
Mts R268A
IP:FLAG
WB:HA
100
70
Wrd
Wdb
IP:FLAG
WB:FLAG
35
Mts WT/R268A
GFP
WB: HA
100
70
Wrd
Wdb
WB: Tubulin
55
Tubulin
D
Relative binding of Wrd to Mts
1.5
1.0
0.5
0.0
*
Mts WT
Mts R268A
E
Relative binding of Wdb to Mts
1.5
1.0
0.5
0.0
Mts WT
Mts R268A

Figure EV5. Validation of dsRNAs and Mts^{R268A}.

(**A**) Validation of *wdb* and *wrd* dsRNA efficiency. S2 cells were treated with the indicated dsRNA for 24 h prior to transfection with the indicated constructs. Cells were lysed 48 h after transfection, and lysates were processed and analysed by western blotting using the indicated antibodies. GFP and Tubulin were used as transfection and loading controls, respectively. *wrd* and *wdb* dsRNAs specifically downregulate Wrd and Wdb protein levels. (**B**) Validation of *tws* dsRNA reagents. Since *tws* dsRNA targets the 3'UTR of the *tws* gene, RT-PCR was conducted on lysates from S2 cells treated with either *lacZ* dsRNA or *tws* dsRNA. RT-PCR reactions were run on a 2% agarose gel. *tws* dsRNA resulted in a reduction in *tws* RNA levels. *rp49* was used as a loading control. (**C–E**) Effect of R286A mutation on the Mts:Wrd and Mts:Wdb interactions. S2 cells were transfected with the indicated constructs. Following lysis, FLAG-tagged proteins were purified using FLAG-agarose beads. Lysates and eluates were processed for Western blot analysis with the indicated antibodies. Tubulin was used as a loading control. (**D**, **E**) Bar charts depicting the levels of either Wrd (**D**) or Wdb (**E**) bound to Mts^{R268A} relative to Mts^{WT} per experiment. Significance was assessed by a paired *t* test, and only significant comparisons were shown. $n = 3$ independent experiments. $^*P < 0.05$. Exact *P* values for all comparisons are listed in Dataset EV1. Source data are available online for this figure.

 © The Author(s)

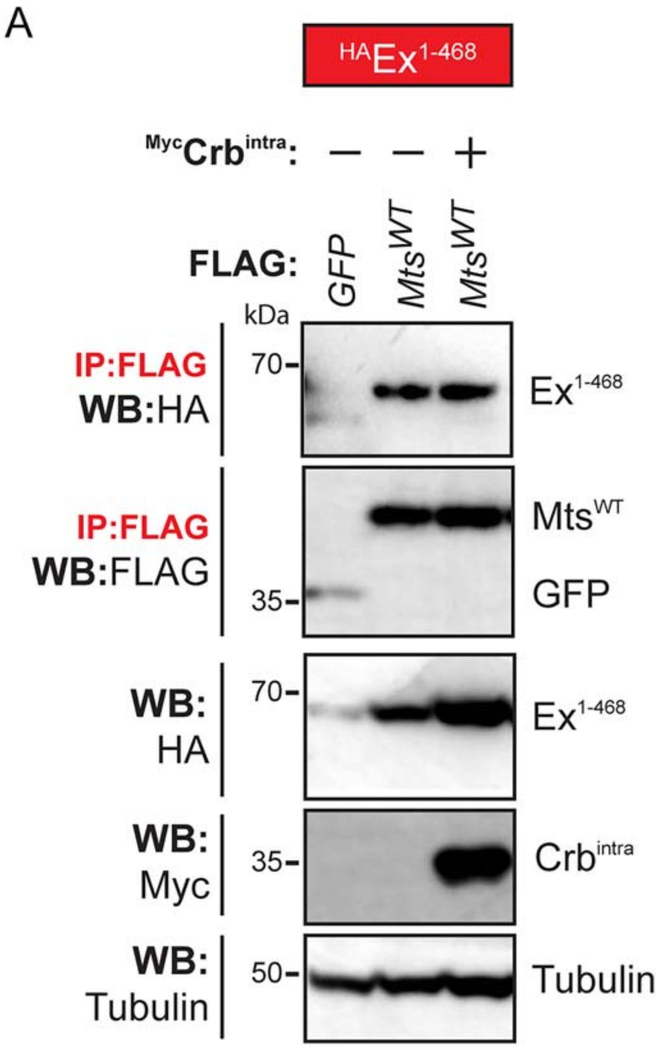

Figure EV6. Mts binds Ex in the absence or presence of Crb expression.

(**A**) Effect of Crb on the Mts:Ex interaction. S2 cells were transfected with the indicated constructs and lysed 48 h after transfection. FLAG-tagged proteins were purified from lysates using FLAG-agarose beads. Lysates and FLAG immunoprecipitates were processed for immunoblot analysis using the indicated antibodies. Tubulin was used as a loading control. Mts co-immunoprecipitated with $Ex^{1\text{-}468}$ in the presence and absence of Crb^{intra}. Source data are available online for this figure.

© The Author(s)
