# Supplementary material for: PP2A phosphatase regulates Hippo signalling in dual manner
Source: EMBO J. 2026 Jul 7;45(16):5943–71. doi: 10.1038/s44318-026-00850-9 (PMC13482807; doi:10.1038/s44318-026-00850-9)
Supplement: Supplementary file 2 [file 44318_2026_850_MOESM1_ESM.pdf]

THE EMBO JOURNAL

PP2A phosphatase regulates Hippo signalling in dual manner

Aashika Sekar, Alberto Rizzo, Elodie Sins, Alexander Fulford, Lucy Silcock, and Paulo Ribeiro

Corresponding authors: Paulo Ribeiro (p.baptista-ribeiro@qmul.ac.uk) , Aashika Sekar (aashika.sekar@crick.ac.uk)

Review Timeline:

Transferred from Review Commons:	19th Mar 25
Editorial Decision:	31st Mar 25
Revision Received:	12th Apr 26
Editorial Decision:	15th Jun 26
Revision Received:	20th Jun 26
Accepted:	24th Jun 26

Editor: Daniel Klimmeck

Transaction Report:

Review COMMONS

This manuscript was transferred to The EMBO Journal following peer review at Review Commons.

(Note: With the exception of the correction of typographical or spelling errors that could be a source of ambiguity, letters and reports are not edited. Depending on transfer agreements, referee reports obtained elsewhere may or may not be included in this compilation. Referee reports are anonymous unless the Referee chooses to sign their reports.)

Review #1

1. Evidence, reproducibility and clarity:

Evidence, reproducibility and clarity (Required)

The paper nicely shows that PP2A antagonizes Crb-dependent and Crb-independent phosphorylation and degradation of Expanded (Ex), in cell culture and in wing discs. The authors focus on the Mts catalytic subunit of PP2A, but also demonstrate the involvement of the Wrd and Tws B regulatory subunits. They also show via use of transcriptional reporters that PP2A directly affects Hpo signaling in vivo. Finally, they show a potential role for Merlin and Kibra in regulating Ex levels, and that Kib binds to Mts and Wrd. The experiments are on the whole well executed and quantified.

Major comments:

1. I am not convinced that the authors can entirely rule out a role for the STRIPAK complex. Mutation of MtsR268A reduces binding of Wrd by 60% and abrogates the effect of Mts on Ex. However mutation of MtsL186A reduces binding of Cka by less than 50% and doesn't disrupt Mts regulation of Ex. Perhaps Cka is more abundant than Wrd, and 50% of Mts/Cka complex is more than sufficient for it to carry out its enzymatic function. I also note that in Fig 1H, Ex levels in Crb/Mts+Cka RNAi appear to be intermediate between those in Crb and Crb/Mts. Ideally this would be quantified. Similarly in 4J, mtsL186A (while not significant) appears intermediate between mtsH118N and mts-WT. What is the actual P value for the comparison to Mts-WT? In any case I would suggest the authors tone down these conclusions.
2. I also found it rather confusing that the authors discuss the Cka B subunit in the context of the STRIPAK complex in Figure 1, then don't look at the other B subunits until Figures 3/4. In my opinion, it would be easier to follow the flow of the manuscript if the authors discussed Crb-dependent and independent regulation of Ex, then the roles of Gish/CKI, then the role of the B subunits including Cka. In this context, it would also be interesting to see if there was any redundancy between Cka and Wrd - have the authors tried any double knockdown experiments (with appropriate controls for RNAi dosage)?
3. The authors examine Crb-independent Ex regulation in the wing disc, which appears to be wing discs that do not overexpress Crb. I would expect that wing discs do express Crb - or is this not the case? Please clarify whether this is in the absence of Crb, or the absence of overexpressed Crb.
4. I was confused by the section 'CKIs and Slmb regulate Ex proteostasis via the 452-457 Slmb consensus sequence'. The authors conclude that 'these results show that the machinery that facilitates Crb-mediated Ex phosphorylation and degradation is also partly involved in the Crb-independent regulation of Ex protein stability.' However, I had concluded the opposite, as it appeared that Slimb and gish RNAi only affected Ex1-468, and similarly Slmb only affected Ex1-468, but not Ex1-450 (which in the previous section was shown to be regulated by Mts independent of Crb). Please could the authors explain/clarify this.
5. The regulation of Ex by Merlin and Kibra is potentially interesting, but a bit preliminary. This part of the manuscript could be strengthened by showing for example if Mts or Wrd knockdown

affects the stabilization of Ex by Kib.

Minor comments:

1. The Introduction gives a quite comprehensive review of known interactions between STRIPAK, Expanded and Hippo pathway components. However, it is hard to keep track of all the components and interactions if you are not deeply into the field. To improve accessibility, I would suggest a summary diagram of the key interactions (currently the manuscript has no introductory figures at all!) and if possible the authors might consider whether there are details they could leave out or which could just be mentioned as necessary in the results sections.
2. Could the authors show a shorter exposure of the Ex blot in Figure 1A, in order to better visualize the loss of band shift?
3. Line 307 '(Fig. 1B,D,G,I)' the call-out to Fig.1I appears to be in strike-through font, presumably because 1I shouldn't be cited here? It also looks like Fig.1I is wrongly cited on line 342 as that sentence only describes action of L168A in wing discs. I think a sentence describing the experiment in Fig.1I is missing?
4. Line 355 ambiguous, should this read low expression of Crb in S2 cells?
5. Line 369 reads 'PP2A was able to stabilize full-length Ex', Mts-WT would be more precise.
6. The blot in panel 2O is mislabeled Ex1-468, I think this should be Ex1-450.
7. The nomenclature of 'Mts-WT' for their own transgene and 'Mts-BL' for the Bloomington transgene. is confusing, as both are, I believe, wild type. Maybe leave this detail for the M&M, at least if the authors believe there is no difference in behavior.
8. Figure S6 appears to be missing from the uploaded version.
9. Lines 480-481: 'Using co-IP analyses, we observed that Mts interacts with Ex, both in the presence and absence of Crbintra.' No figure call-out is given for this statement, and I can't see the data anywhere, but from the figure legends it seems to be in the missing Fig.S6? And everything that follows in this paragraph should have call-outs for Fig.4K?
10. Lines 503-504: 'we found that Kib associated with Mts (Fig. 5C)' - Fig.5B?
11. Lines 504-505: 'no interaction was observed between Mts and Mer (Fig.5B)' - Fig.5C?
12. In Figure 6G, authors note that 'the mean diap1GFP4.3 levels of MtsWT+Crb-Intra were lower than those of Crb-Intra, this difference was not statistically significant when all genotypes were included in the comparisons, but only when the Control, crbintra and mtsWT+crbintra conditions were considered.' It might be useful to have a table showing the actual P values of all the comparisons (or maybe better still just put actual P values on the graphs?). Sometimes an arbitrary cut-off of 0.05 for significant can be misleading.

Referees cross-commenting

*this session contains comments from ALL the reviewers"
Rev1

All comments look very fair and we seem to have similar views, so nothing further to add on our part.
Rev 2

Agreed. We think the reviews provide a consistent guide for revisions/additions that would

enhance impact of the studies and rigor of the conclusions.
Rev 3

I also find the other reviewers' comments to be fair. Major issues that stick out are:
1. is the effect really independent of STRIPAK?
2. do the effects seen on ectopic Ex1-468 apply to endogenous Ex?

A relatively simple experiment could possibly address both issues. If the model is correct and PP2A can target both Hippo and Ex using different adaptor proteins, then we would expect modulating the levels of Tws and Wrd adaptors to influence Ex stability, but not Hpo phosphorylation. Could the authors test this hypothesis in vivo, looking at the endogenous proteins?

Do the other reviewers think that this would be a fair experiment to ask for?
Rev 1
With regard to points of rev 3, I think it's perfectly fair to ask for more data to support the conclusions, and specifically what they suggest regarding separating effects on Hippo and Ex is obviously helpful.
The broader question (which I'm unsure how to address in the context of Review Commons) is 'what is necessary for publication' as that depends on where the authors aspire to publish.
I would be fine with the authors softening their conclusions and adding caveats instead of adding more data. However, it is also true that adding more data would increase the certainty of their conclusions and lead to a more valuable publication.
This is a question for the editor of the journal that they finally submit to, but I'm not sure as reviewers how we lay out these options. Do we add an extra review comment saying either (i) soften conclusions for less valuable paper, (ii) add more data for more valuabe paper, and then leave the authors to argue the point with an editor. In particular the STRIPAK dependence was raised in 2 reviews, so an editor would probably pick up on this.
Rev 2
In past reviews for Review Commons, we've distinguished between three levels of review requests: (1) what is minimally necessary to publish (ie egregious gaps); (2) what would enhance confidence in the conclusions, and finally (3) what, if anything, would turn it into a high impact/visibility paper.

I think most of our suggestions for additional expts fall into category #2 as "either tone down the language or add expt X".
Rev 1
That sounds reasonable.

2. Significance:

Significance (Required)

The Hippo signaling pathway is a conserved regulator of tissue growth, and understanding how this pathway is activated and modulated is of great importance. Levels of the upstream activator Expanded are known to be regulated by phosphorylation/degradation, but whether

dephosphorylation of Ex is important for growth control has not been widely investigated. This paper utilizes cell culture and the fruit fly model organism to provide clear evidence for a role for PP2A in regulation of Ex levels, independent of its known role in regulating phosphorylation of Hpo. It will therefore be of interest to biologists working in the fields of growth control and tissue homeostasis.

Expertise: developmental biology, Drosophila research, cell biology

3. How much time do you estimate the authors will need to complete the suggested revisions:

Estimated time to Complete Revisions (Required)

(Decision Recommendation)

Between 1 and 3 months

4. *Review Commons* values the work of reviewers and encourages them to get credit for their work. Select 'Yes' below to register your reviewing activity at Web of Science Reviewer Recognition Service (formerly Publons); note that the content of your review will not be visible on Web of Science.

Web of Science Reviewer Recognition

Yes

Review #2

1. Evidence, reproducibility and clarity:

Evidence, reproducibility and clarity (Required)

Summary: The authors show that the protein phosphatase PP2A antagonizes Crb-mediated phosphorylation and subsequent degradation of Expanded in vivo. Using Drosophila imaginal wing discs and the GAL4-UAS system, the authors provide evidence that the PP2A holoenzyme dephosphorylates Ex, stabilizing its protein levels, in a manner independent of the STRIPAK complex and identifies Wrd as a key regulatory subunit of PP2A in this process. Importantly, the

study also shows that PP2A stabilizes Ex protein levels independent of Crb-driven phosphorylation and that, via this stabilization, PP2A activates Hpo pathway signaling to repress transcriptional targets of Yki.

Major comments: Overall, the study is strong, and the conclusions are supported by the data. The data does largely lean on overexpression models in the wing disc and it would strengthen the biological relevance to include genomic alleles (i.e., do Ex-GFP levels go down in PP2A/mts mutant clones?). Materials and methods are thoroughly presented, and statistical analyses are adequate. OPTIONAL: While not necessarily required for publication, note that full in vivo confirmation would require altering the PP2A target sites in Ex by generating phospho-deficient and phospho-mimetic versions and seeing if they match the model. This would push the conclusions to the highest degree of confidence and rigor.

Minor comments: Text and figures are clear and accurate. It may be helpful to include a modified version of the Mts mutants table in SF1 in a main figure for easier reference but is not necessary.

2. Significance:

Significance (Required)

The studies strengths include biochemical and in vivo validation of the effect of PP2A and its various regulatory subunits on Ex phosphorylation and stabilization. The study very methodically parses out the context in which PP2A is stabilizing Ex (i.e., both in the context of Crb stimuli and independently, and it does so independently of the STRIPAK complex). As noted previously, recapitulating the major results in clones using genomic alleles would strengthen the biological relevance. The study advances our understanding of mechanisms tightly controlling downstream transcriptional outputs of the Hpo pathway via regulating Ex protein stability/turnover. Though the primary audience may be those well-versed in the Hpo field and Drosophila genetics, the implications for the research are broad.

3. How much time do you estimate the authors will need to complete the suggested revisions:

Estimated time to Complete Revisions (Required)

(Decision Recommendation)

Between 1 and 3 months

4. *Review Commons* values the work of reviewers and encourages them to get credit for their work. Select 'Yes' below to register your reviewing activity at [Web of Science]

Reviewer Recognition Service (formerly Publons); note that the content of your review will not be visible on Web of Science.

Web of Science Reviewer Recognition

Yes

Review #3

1. Evidence, reproducibility and clarity:

Evidence, reproducibility and clarity (Required)

The authors hypothesized that Crb mediated Ex phosphorylation and degradation, that they previously established, should be countered and set on to identify the phosphatase involved. Surprisingly, they find that Mts, the catalytic subunit of PP2A, counters the effect of ectopically expressed intracellular domain of Crb on Ex stability. This was surprising because PP2A and the STRIPAK complex was shown to counter Hippo activity previously, suggesting that PP2A would inject both positive and negative inputs into Hpo activity. The title reflects this finding.

Overall, the experiments are well controlled and are of high quality. I especially appreciate the effort to show results of parallel experiments both in S2 cells and in vivo in wing discs.

The manuscript convincingly demonstrates that Mts expression stabilizes Ex1-468::GFP in the presence or absence of ectopic Crb-intra. This effect is mainly mediated by the Wrd adaptor subunit, and requires the catalytic activity of Mts. However, results shown in Fig4K highlights the Tws adaptor as the main one that binds to and stabilizes Ex in S2 cells, in the presence or absence of Crb-intra expression. This is slightly at odds with Wrd-RNAi experiments nicely reversing the effects of Crb-intra expression.

The manuscript is not easy to read given the vast amount of data using many different constructs, but there is little the authors can do about it as the story is complex and layered.

The argument that the effects of Mts are independent of the STRIPAK complex is less convincing. This conclusion is based on Mts-L186A mutant which should not bind Cka which is the PP2A adaptor subunit found in the STRIPAK complex. Fig S3F and G show that Cka binding to Mts is reduced by half when Mts-L186A mutant is expressed in lieu wt Mts. Consistent with this in Fig1F rescue of Ex degradation by Mts-L186A is half as effective as the rescue seen in 1F by the wt Mts. Towards the same argument, data shown on S3A-D is deemed inconclusive based on quantification in S3E which does not reflect the clear reduction in Ex that is seen in S3B. Hence FigS3 is in favour of Cka4 being involved in the rescue effect.

In Figures 5A and 3A, Crb-intra expression does not destabilize Ex1-468::GFP, why is that?

The authors connect effects on Ex stability to the influence on Hippo pathway activity in Fig 6, which is a very nice touch.

Finally, I wonder whether the dual effect of PP2A on Hippo activity (inhibiting Hippo and stabilizing Ex) could be a single effect. I am guessing the Ex1-468::GFP construct, having its own regulatory elements, would act independently of the transcriptional activity of Hippo. However, I was not able to find this demonstrated in the literature. Can the authors show that? For example, make hpo or wts mutant clones in the presence of the Ex1-468::GFP construct. Otherwise, an alternative explanation could be that PP2A, with its various adaptor subunits, counters Hippo activity which translates into higher levels of expanded transcription and Ex protein production. It was also demonstrated that there are higher levels of Crb in hippo mutants likely due to the expansion of the apical domain. This would be consistent with the stabilized Crb-intra seen in Figures 1A&3A upon Mts expression. Stabilization of Crb upon Mts expression (not commented on in the manuscript) is very interesting as extra Crb should further push the balance towards Ex degradation but Mts seems to be able to reverse the effect. I agree that this alternative explanation may be far-fetched, yet it is also easily tested, and would greatly simplify the model put forward.

Finally, if indeed various PP2A complexes, depending on the adaptor subunits they contain, have a range of effects on Ex stability and Hippo pathway activity, this brings in the question of what regulates the availability of various adaptor subunits and the PP2A complexes they form? The question is outside the scope of the manuscript but it is worth discussing.

2. Significance:

Significance (Required)

A vast amount of data is presented in both in vivo and in vitro settings. The study uses biochemical and genetic approaches and combines them aptly.

I think the findings showing multiple and various effects on PP2A on the same pathway would be of higher interest to the PP2A enthusiasts than the Hippo researchers.

3. How much time do you estimate the authors will need to complete the suggested revisions:

Estimated time to Complete Revisions (Required)

(Decision Recommendation)

Between 1 and 3 months

4. *Review Commons* values the work of reviewers and encourages them to get credit for their work. Select 'Yes' below to register your reviewing activity at Web of Science Reviewer Recognition Service (formerly Publons); note that the content of your review will not be visible on Web of Science.

Web of Science Reviewer Recognition

No

Revision Plan

Manuscript number: RC-2024-02819
Corresponding author(s): Paulo S Ribeiro

[The "revision plan" should delineate the revisions that authors intend to carry out in response to the points raised by the referees. It also provides the authors with the opportunity to explain their view of the paper and of the referee reports.

The document is important for the editors of affiliate journals when they make a first decision on the transferred manuscript. It will also be useful to readers of the reprint and help them to obtain a balanced view of the paper.

If you wish to submit a full revision, please use our "[Full Revision](#)" template. ***It is important to use the appropriate template to clearly inform the editors of your intentions.****]*

1. General Statements [optional]

This section is optional. Insert here any general statements you wish to make about the goal of the study or about the reviews.

We thank the reviewers for their insightful comments and suggestions. In this revision plan, we will further investigate PP2A-mediated post-translational stabilisation of Ex post-translational is indeed independent of the STRIPAK complex. Additionally, we plan to extend upon how Kib and PP2A interact to regulate Ex stability.

2. Description of the planned revisions

Insert here a point-by-point reply that explains what revisions, additional experimentations and analyses are planned to address the points raised by the referees.

1. To further investigate whether PP2A can indeed stabilise Ex independently of the STRIPAK complex we will conduct the following experiments in response to the comments from Reviewers 1 and 3:
   1.1. Test whether knocking down other components of the STRIPAK complex such as FGOP2 and Mob4 affects the ability of Mts to stabilise Ex degradation in the presence or absence of Crb^{intra} *in vitro* using S2 cells. If we do observe any effect, we will also test whether knocking these components in the posterior compartment of the wing disc also has an effect on the Ex stability reporter levels.
   1.2. Since the reviewers raised the point that the MtsL186A mutant results in 50% reduction in binding with Cka and that a 50% reduction in the Mts/Cka complex may still be sufficient to stabilise Ex levels. To address this, we will knock down either Wrd or Cka and test whether this affects the ability of MtsL186A to stabilise Ex both in the

presence/absence of Crb^{intra}. This will test whether the stabilisation of Ex by MtsL186A can be attributed to the function of the $MtsL186A^{Cka}$ holoenzyme or the $MtsL186A^{Wrd}$ holoenzyme. We will test this both *in vitro* and *in vivo*.

2. Based on Reviewer 1's suggestion we will undertake knock down both Cka and Wrd to test for any redundancy between Cka and Wrd using S2 cells.
3. As per the suggestion by Reviewer 1, we will further characterise the interaction between Kib and Mts in stabilising Ex. We will test whether Kib can stabilise Ex when either Mts or Wrd is knocked down. We will also test whether Kib can stabilise Ex in the absence of ectopic Crb expression *in vivo* and whether this is indeed dependent on the Wrd subunit.
4. Reviewer 3 suggested that Mts may potentially be involved in regulating Crb^{intra} levels. To test this, we will test whether overexpression of various doses of either Mts^{WT} or Mts^{H118N} affects the stability of Crb^{intra} using S2 cells.
5. Reviewer 3 suggested that there is insufficient evidence to show that the ubi-$Ex^{1\text{-}468}$::GFP reporter is truly independent of Hpo pathway activity. Since the reporter is under the control of the *ubiquitin 63E* promoter as opposed to the endogenous promoter, we do not envisage that its transcription is regulated by Yki. Indeed, a similar method of decoupling potential transcriptional and post-translational effects of Hpo signalling has been successfully used in studies that have focused on other Hpo pathway components, such as Kibra (Tokamov et al., 2021) and Salvador (Aerne et al., 2015). The reviewer suggests that we should assess the effect of *hpo* or *wts* mutant clones and determine of these affect the levels of the ubi-$Ex^{1\text{-}468}$::GFP reporter. However, we believe this may lead to results that will be difficult to interpret. Although *hpo* or *wts* clones are expected to result in higher Yki activity, they will also remove Hpo or Wts function, and these proteins may be involved in the molecular mechanisms that regulate Ex protein stability. Therefore, as an alternative approach to assess the impact of Hpo signalling on the Ex reporter, we will perform RT-PCR experiments to monitor the transcriptional regulation of the transgenic reporter in the presence or absence of Yki overexpression.

3. Description of the revisions that have already been incorporated in the transferred manuscript

Please insert a point-by-point reply describing the revisions that were already carried out and included in the transferred manuscript. If no revisions have been carried out yet, please leave this section empty.

Reviewer 1:

Major Comments

(1) I also note that in Fig 1H, Ex levels in Crb/Mts+Cka RNAi appear to be intermediate between those in Crb and Crb/Mts. Ideally this would be quantified.

A quantification for Ex levels between Mts^{WT}+Crb^{intra}+lacZ-RNAi and Mts^{WT}+Crb^{intra}+cka-RNAi is now presented in Fig. 1J.

(3) The authors examine Crb-independent Ex regulation in the wing disc, which appears to be wing discs that do not overexpress Crb. I would expect that wing discs do express Crb - or is this not the case? Please clarify whether this is in the absence of Crb, or the absence of overexpressed Crb.

This is now clarified in the text Line 358.

(4) I was confused by the section 'CKIs and Slmb regulate Ex proteostasis via the 452-457 Slmb consensus sequence'. The authors conclude that 'these results show that the machinery that facilitates Crb-mediated Ex phosphorylation and degradation is also partly involved in the Crb-independent regulation of Ex protein stability.' However, I had concluded the opposite, as it appeared that Slimb and gish RNAi only affected Ex1-468, and similarly Slmb only affected Ex1-468, but not Ex1-450 (which in the previous section was shown to be regulated by Mts independent of Crb). Please could the authors explain/clarify this.

This is now clarified in the text Lines 387-388 and Lines 405-406.

Minor Comments:

(1) The Introduction gives a quite comprehensive review of known interactions between STRIPAK, Expanded and Hippo pathway components. However, it is hard to keep track of all the components and interactions if you are not deeply into the field. To improve accessibility, I would suggest a summary diagram of the key interactions (currently the manuscript has no introductory figures at all!) and if possible the authors might consider whether there are details they could leave out or which could just be mentioned as necessary in the results sections.

An introductory figure has now been added as Fig. 1A.

(2) Could the authors show a shorter exposure of the Ex blot in Figure 1A, in order to better visualize the loss of band shift?

A shorter exposure of the Ex blot has now been added to the Fig. 1B (previously Fig. 1A).

(3) Line 307 '(Fig. 1B,D,G,I)' the call-out to Fig.1I appears to be in strike-through font, presumably because 1I shouldn't be cited here? It also looks like Fig.1I is wrongly cited on line 342 as that sentence only describes action of L168A in wing discs. I think a sentence describing the experiment in Fig.1I is missing?

The Figures have now been cited appropriately. Fig. 1J (previously Fig. 1I) is now referred to in Line 336.

(4) Line 355 ambiguous, should this read low expression of Crb in S2 cells?

This has now been changed from extremely low expression to low expression.

(5) Line 369 reads 'PP2A was able to stabilize full-length Ex', Mts-WT would be more precise.

This has now been changed to Mts^{WT} was able to stabilise full-length Ex.

(6) The blot in panel 2O is mislabeled Ex1-468, I think this should be Ex1-450.

The blot in panel 2O is now correctly labelled as Ex$^{1\text{-}450}$.

(8) Figure S6 appears to be missing from the uploaded version.

Fig. S6 has been appended.

(9) Lines 480-481: 'Using co-IP analyses, we observed that Mts interacts with Ex, both in the presence and absence of Crbintra.' No figure call-out is given for this statement, and I can't see the data anywhere, but from the figure legends it seems to be in the missing Fig.S6? And everything that follows in this paragraph should have call-outs for Fig.4K?

Fig. S6 has now been appended and the call-outs to Fig. 4K have been added to in the paragraph Line 475-490.

(10) Lines 503-504: 'we found that Kib associated with Mts (Fig. 5C)' - Fig.5B?

This has now been changed.

(11) Lines 504-505: 'no interaction was observed between Mts and Mer (Fig.5B)' - Fig.5C?

This has now been changed.

(12) In Figure 6G, authors note that 'the mean diap1GFP4.3 levels of MtsWT+Crb-Intra were lower than those of Crb-Intra, this difference was not statistically significant when all genotypes were included in the comparisons, but only when the Control, crbintra and mtsWT+crbintra conditions were considered.' It might be useful to have a table showing the actual P values of all the comparisons (or maybe better still just put actual P values on the graphs?). Sometimes an arbitrary cut-off of 0.05 for significant can be misleading.

We have now added the actual p-values for those >0.05 to the graph.

4. Description of analyses that authors prefer not to carry out

Please include a point-by-point response explaining why some of the requested data or additional analyses <u>might not be necessary or cannot be provided within the scope of a revision</u>.

This can be due to time or resource limitations or in case of disagreement about the necessity of such additional data given the scope of the study. Please leave empty if not applicable.

Reviewer #2:

Major comments: Overall, the study is strong, and the conclusions are supported by the data. The data does largely lean on overexpression models in the wing disc and it would strengthen the biological relevance to include genomic alleles (i.e., do Ex-GFP levels go down in PP2A/mts mutant clones?).

We agree with the reviewer and indeed have tried to undertake MARCM experiments with mts null mutant clones. However, since mts is an essential gene, even when Mts^{WT} was expressed in the presence of mts mutant, we were only able to obtain few single cell clones, which was difficult to analyse. Hence, clonal analysis using mts mutant clones will not be feasible.

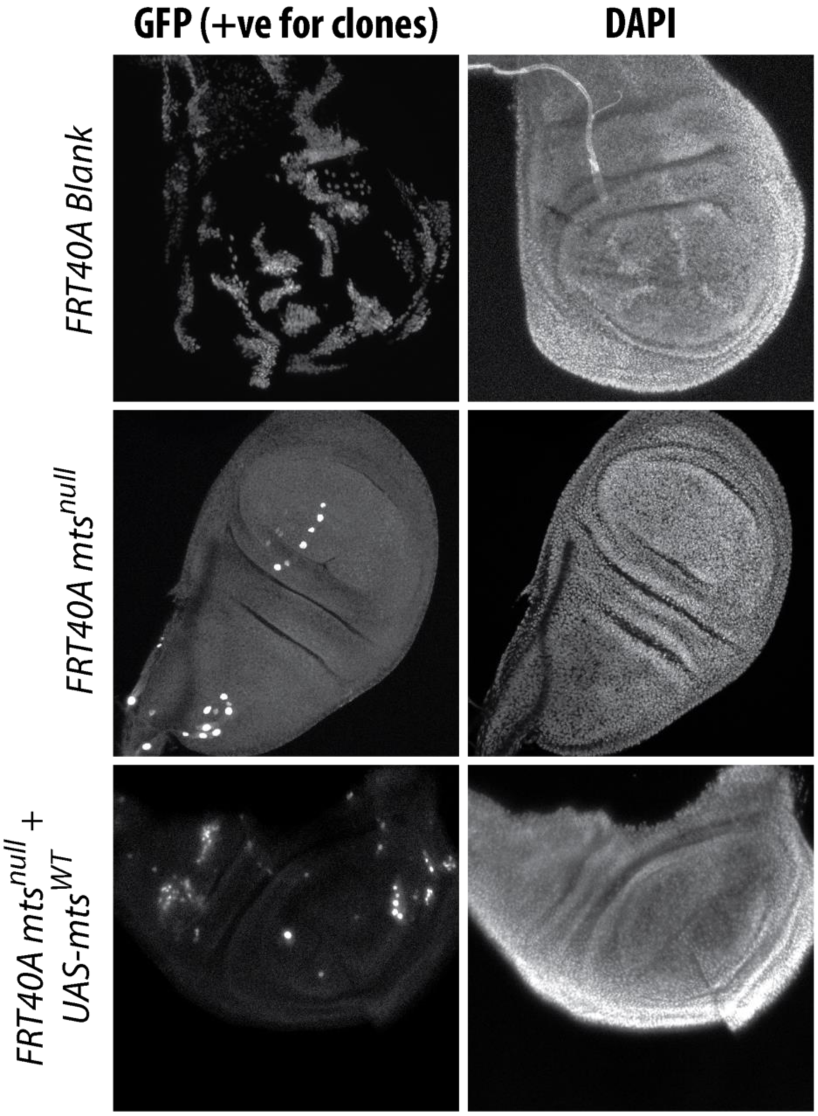

Dear Dr Ribeiro,

Thank you for transferring your manuscript EMBOJ-2025-120833-T | [RC-2024-02819] with Review Commons referee reports and responses to The EMBO Journal, and also for your patience with our feedback at this time of the year. I have now carefully assessed your revision plan and rebuttal to the referees at Review Commons, and in addition discussed this in the editorial team.

We acknowledge that you are planning a substantial experimental revision to complement your study and address the critique raised by the referees and consider your response to be sensible. We can thus invite you to revise your study along the lines sketched in your outline for further consideration by the EMBO Journal.

Please feel free to contact me if you have any questions or need further input on the referee comments.

We generally allow three months as standard revision time. As a matter of policy, competing manuscripts published during this period will not negatively impact on our assessment of the conceptual advance presented by your study. However, we request that you contact the editor as soon as possible upon publication of any related work, to discuss how to proceed. Should you foresee a problem in meeting this three-month deadline, please let us know in advance and we may be able to grant an extension.

When submitting your revised manuscript, please carefully review the instructions below.

Please feel free to approach me any time should you have additional questions related to this.

Thank you for the opportunity to consider your work for publication.

I look forward to your revision.

Kind regards,

Daniel Klimmeck

Daniel Klimmeck, PhD
Senior Editor
The EMBO Journal

Instruction for the preparation of your revised manuscript:

1) a .docx formatted version of the manuscript text (including legends for main figures, EV figures and tables). Please make sure that the changes are highlighted to be clearly visible.

2) individual production quality figure files as .eps, .tif, .jpg (one file per figure).

3) a .docx formatted letter INCLUDING the reviewers' reports and your detailed point-by-point response to their comments. As part of the EMBO Press transparent editorial process, the point-by-point response is part of the Review Process File (RPF), which will be published alongside your paper.

4) a complete author checklist, which you can download from our author guidelines (https://wol-prod-cdn.literatumonline.com/pb-assets/embo-site/Author Checklist%20-%20EMBO%20J-1561436015657.xlsx). Please insert information in the checklist that is also reflected in the manuscript. The completed author checklist will also be part of the RPF.

5) Please note that all corresponding authors are required to supply an ORCID ID for their name upon submission of a revised manuscript.

6) It is mandatory to include a 'Data Availability' section after the Materials and Methods. Before submitting your revision, primary datasets produced in this study need to be deposited in an appropriate public database, and the accession numbers and database listed under 'Data Availability'. Please remember to provide a reviewer password if the datasets are not yet public (see https://www.embopress.org/page/journal/14602075/authorguide#datadeposition).
In case you have no data that requires deposition in a public database, please state so in this section. Note that the Data Availability Section is restricted to new primary data that are part of this study.
*** Note - All links should resolve to a page where the data can be accessed. ***

7) Our journal encourages inclusion of *data citations in the reference list* to directly cite datasets that were re-used and obtained from public databases. Data citations in the article text are distinct from normal bibliographical citations and should directly link to the database records from which the data can be accessed. In the main text, data citations are formatted as follows: "Data ref: Smith et al, 2001" or "Data ref: NCBI Sequence Read Archive PRJNA342805, 2017". In the Reference list, data citations must be labeled with "[DATASET]". A data reference must provide the database name, accession number/identifiers and a resolvable link to the landing page from which the data can be accessed at the end of the reference. Further instructions are available at .

8) At EMBO Press we ask authors to provide source data for the main and EV figures. Our source data coordinator will contact you to discuss which figure panels we would need source data for and will also provide you with helpful tips on how to upload and organize the files.

Numerical data can be provided as individual .xls or .csv files (including a tab describing the data). For 'blots' or microscopy, uncropped images should be submitted (using a zip archive or a single pdf per main figure if multiple images need to be supplied for one panel). Additional information on source data and instruction on how to label the files are available at .

9) We replaced Supplementary Information with Expanded View (EV) Figures and Tables that are collapsible/expandable online (see examples in https://www.embopress.org/doi/10.15252/embj.201695874). A maximum of 5 EV Figures can be typeset. EV Figures should be cited as 'Figure EV1, Figure EV2" etc. in the text and their respective legends should be included in the main text after the legends of regular figures.

- For the figures that you do NOT wish to display as Expanded View figures, they should be bundled together with their legends in a single PDF file called *Appendix*, which should start with a short Table of Content. Appendix figures should be referred to in the main text as: "Appendix Figure S1, Appendix Figure S2" etc. See detailed instructions regarding expanded view here: .

- Additional Tables/Datasets should be labelled and referred to as Table EV1, Dataset EV1, etc. Legends have to be provided in a separate tab in case of .xls files. Alternatively, the legend can be supplied as a separate text file (README) and zipped together with the Table/Dataset file.

10) When assembling figures, please refer to our figure preparation guideline in order to ensure proper formatting and readability in print as well as on screen:
http://bit.ly/EMBOPressFigurePreparationGuideline

Please remember: Digital image enhancement is acceptable practice, as long as it accurately represents the original data and conforms to community standards. If a figure has been subjected to significant electronic manipulation, this must be noted in the figure legend or in the 'Materials and Methods' section. The editors reserve the right to request original versions of figures and the original images that were used to assemble the figure.

11) For data quantification: please specify the name of the statistical test used to generate error bars and P values, the number (n) of independent experiments (specify technical or biological replicates) underlying each data point and the test used to calculate p-values in each figure legend. The figure legends should contain a basic description of n, P and the test applied. Graphs must include a description of the bars and the error bars (s.d., s.e.m.).

Please remember: Digital image enhancement is acceptable practice, as long as it accurately represents the original data and conforms to community standards. If a figure has been subjected to significant electronic manipulation, this must be noted in the figure legend or in the 'Materials and Methods' section. The editors reserve the right to request original versions of figures and the original images that were used to assemble the figure.

Further information is available in our Guide For Authors: https://www.embopress.org/page/journal/14602075/authorguide

We realize that it is difficult to revise to a specific deadline. In the interest of protecting the conceptual advance provided by the work, we recommend a revision within 3 months (29th Jun 2025). Please discuss the revision progress ahead of this time with the editor if you require more time to complete the revisions.

***

Rev_Com_number: RC-2024-02819
New_manu_number: EMBOJ-2025-120833-T
Corr_author: Ribeiro
Title: A Dual Role for the PP2A Phosphatase in Hippo Signalling Regulation

"A Dual Role for the PP2A Phosphatase in Hippo Signalling Regulation"
Sekar *et al.* EMBOJ-2025-120833R

We thank the reviewers for their insightful comments and for their suggestions which have helped us to substantially improve our manuscript. We summarise our main new findings before providing a point by point reply to the reviewers' comments. For some of the comments raised, we also include data in the rebuttal letter which, if so required by the reviewers, we can include in the revised manuscript.
Based on the reviewers' suggestions, we include the following new data in the revised manuscript:

1) **Figure 3B** includes quantification of blots showing that *wrd*-RNAi prevents PP2A-dependent stabilisation of Ex in presence of Crb^{intra}.
2) **Figures 3K, L, O** show that knocking down Wrd in the context of Mts^{L186A} expression (mutant that reduces binding to Cka) abrogates the stabilisation of Ex in the presence of Crb^{intra}, suggesting that the effect is largely due to the interaction of Mts with Wrd.
3) **Figure 6** includes p-value for comparison between Crb^{intra} and Mts$^{WT/Mutants}$+Crb^{intra}.
4) **Figure S1A** shows that Mts does not regulate Crb^{intra} levels.
5) **Figures S2C-F** show that the *ubi-Ex*$^{1-468}$*::GFP* reporter is indeed independent of Yki transcriptional activity.
6) **Figure S3A** includes quantification of blots showing that *cka*-RNAi does not affect the ability of PP2A to stabilise Ex in the presence of Crb^{intra}.

Below, we provide further details and point by point responses to the reviewer's comments.

Reviewer #1 comments
Significance
The Hippo signaling pathway is a conserved regulator of tissue growth, and understanding how this pathway is activated and modulated is of great importance. Levels of the upstream activator Expanded are known to be regulated by phosphorylation/degradation, but whether dephosphorylation of Ex is important for growth control has not been widely investigated. This paper utilizes cell culture and the fruit fly model organism to provide clear evidence for a role for PP2A in regulation of Ex levels, independent of its known role in regulating phosphorylation of Hpo. It will therefore be of interest to biologists working in the fields of growth control and tissue homeostasis.

Evidence, reproducibility and clarity
The paper nicely shows that PP2A antagonizes Crb-dependent and Crb-independent phosphorylation and degradation of Expanded (Ex), in cell culture and in wing discs. The authors focus on the Mts catalytic subunit of PP2A, but also demonstrate the involvement of the Wrd and Tws B regulatory subunits. They also show via use of transcriptional reporters that PP2A directly affects Hpo signaling in vivo. Finally, they show a potential role for Merlin and Kibra in regulating Ex levels, and that Kib binds to Mts and Wrd. The experiments are on the whole well executed and quantified.
We thank the reviewer for their analysis of the significance of our report and are encouraged by the fact that the reviewer considers that the manuscript includes well executed and quantified experimental approaches.

Reviewer #1 major comments
1: I am not convinced that the authors can entirely rule out a role for the STRIPAK complex. Mutation of MtsR268A reduces binding of Wrd by 60% and abrogates the effect of Mts on Ex. However mutation of MtsL186A reduces binding of Cka by less than 50% and doesn't disrupt Mts regulation of Ex. Perhaps Cka is more abundant than Wrd, and 50% of Mts/Cka complex is more than sufficient for it to carry out its enzymatic function. I also note that in Fig 1H, Ex levels in Crb/Mts+Cka RNAi appear to be intermediate between those in Crb and Crb/Mts. Ideally this would be quantified. Similarly in 4J, mtsL186A (while not significant) appears intermediate between mtsH118N and mts-WT. What is the actual P value for the comparison to Mts-WT?
In any case I would suggest the authors tone down these conclusions.
We appreciate the reviewer's point regarding the effect of the STRIPAK complex and, indeed, the fact that the Mts mutants generated do not entirely block the binding to the regulatory subunits as expected from structural studies complicates the interpretation of the data. To address the concerns raised by the reviewer regarding our interpretation of the role of the STRIPAK complex, we performed experiments where we combined the Mts^{L186A} mutation with depletion of *wrd* using RNAi (Fig. 3K,L,O). Since we have shown that expression of either *wrd* ^{RNAi}+Mts^{WT} or Mts^{R268A} (mutant that affects binding with Wrd) in the presence of Crb^{intra} prevents Mts from stabilising Ex (Fig. 3D-G, J, M, N), we tested whether Mts^{L186A} stabilises Ex via Wrd or whether this is due to its residual binding to Cka. Our data show that depletion of *wrd* abrogates the effect of Mts^{L186A}, strongly suggesting that any effect of the mutant Mts on the regulation of Ex protein levels is

indeed due to its ability to bind Wrd and not due to any residual binding to Cka. If the Mts:Cka complex that can still form when Mts is mutated in R268A had an essential function, we would expect that it would compensate for *wrd RNAi* and this is clearly not the case. We believe this provides strong evidence that our model is correct and that STRIPAK is not essential for this process.

2: I also found it rather confusing that the authors discuss the Cka B subunit in the context of the STRIPAK complex in Figure 1, then don't look at the other B subunits until Figures 3/4. In my opinion, it would be easier to follow the flow of the manuscript if the authors discussed Crb-dependent and independent regulation of Ex, then the roles of Gish/CKI, then the role of the B subunits including Cka. In this context, it would also be interesting to see if there was any redundancy between Cka and Wrd - have the authors tried any double knockdown experiments (with appropriate controls for RNAi dosage)?
We acknowledge the reviewer's comment regarding the discussion of the roles of the PP2A regulatory subunits. We chose to include a discussion of the role of Cka at the very start as, given what is known in the Hippo signalling field, the obvious candidate for a PP2A-mediated regulatory function would be the STRIPAK complex. Therefore, addressing its potential role before exploring other possibilities seemed more logical for us. We thank the reviewer for their suggestion to test any redundancy between Cka and Wrd. Since knocking down Wrd results in a significant decrease in Ex levels and knocking down Cka does not have an effect, a double-knockdown experiment will be difficult to interpret in our system. However, as mentioned above, our results with *wrd*^{RNAi}, Mts^{L186A}, Mts^{R268A} and Mts^{L186A}+*wrd* ^{RNAi} (Fig. 3) demonstrate that Wrd is the main subunit involved in stabilising Ex in the presence of Crb.

3: The authors examine Crb-independent Ex regulation in the wing disc, which appears to be wing discs that do not overexpress Crb. I would expect that wing discs do express Crb - or is this not the case? Please clarify whether this is in the absence of Crb, or the absence of overexpressed Crb.
We apologise for the lack of clarity in this point. Yes, the reviewer is correct that the *in vivo* regulation of Ex protein levels in the wing disc has been assessed in the absence of overexpressed Crb^{intra}, but cells will still express endogenous Crb. This is clarified in Lines 360 and 367.

4: I was confused by the section 'CKIs and Slmb regulate Ex proteostasis via the 452-457 Slmb consensus sequence'. The authors conclude that 'these results show that the machinery that facilitates Crb-mediated Ex phosphorylation and degradation is also partly involved in the Crb-independent regulation of Ex protein stability.' However, I had concluded the opposite, as it appeared that Slimb and gish RNAi only affected Ex1-468, and similarly Slmb only affected Ex1-468, but not Ex1-450 (which in the previous section was shown to be regulated by Mts independent of Crb). Please could the authors explain/clarify this.
We apologise for the lack of clarity regarding this point. We show that Mts can control Ex protein levels in a Crb-independent manner as the protein levels of Ex are elevated in the presence of Mts even when Crb^{intra} is not present. Interestingly, the effect of Mts is seen even when a truncation that lacks the Slimb consensus site is absent (Ex1-450), which suggests that there may be different mechanisms in play. Given their role in the regulation of Ex levels in the presence of Crb^{intra} (Fulford et al. 2019, PMID:31567070) we also tested the role of CKI kinases and Slimb and whether they could also regulate the levels of Ex in the absence of Crb^{intra}. We found that while the CKI kinases and Slimb regulate Ex levels in the absence of Crb^{intra}, this appears to still be dependent on the Slimb degron consensus sequence, as it was not observed when the Ex1-450 truncation was tested. This suggests that Gish/Ck1α/Slmb regulates Ex via the Slmb degron, both in absence and presence of Crb^{intra}. We have now clarified this in the text: Lines 397 and 414-419.

5: The regulation of Ex by Merlin and Kibra is potentially interesting, but a bit preliminary. This part of the manuscript could be strengthened by showing for example if Mts or Wrd knockdown affects the stabilization of Ex by Kib.
In order to build on our in vitro data, we tested the effect of overexpressing Kibra on the levels of the Ex stability reporter (by expressing transgenes in the posterior compartment of the wing, as shown for Mts) (Rebuttal Figure 1). Unfortunately, the wing discs overexpressing Kibra had severe morphological defects in the posterior compartment, making it very challenging to analyse the effect of Kibra on Ex in vivo in these conditions. However, given the known interaction between Crb and Kibra, we believe that the role of Mer and Kibra in regulation of Ex is an interesting addition to our report. We have also attempted to address if the role of Kibra is entirely dependent on the function of Mts. For this, we co-expressed Kibra and Ex in the presence or absence of mts depletion by RNAi (Rebuttal Figure 2). As shown in the figure, depletion of mts largely prevents the Kibra-mediated stabilisation of Ex and an intermediate phenotype is observed, which suggests that Kibra relies on Mts and PP2A to exert its full action on Ex protein levels, but that alternative mechanisms may also be involved.

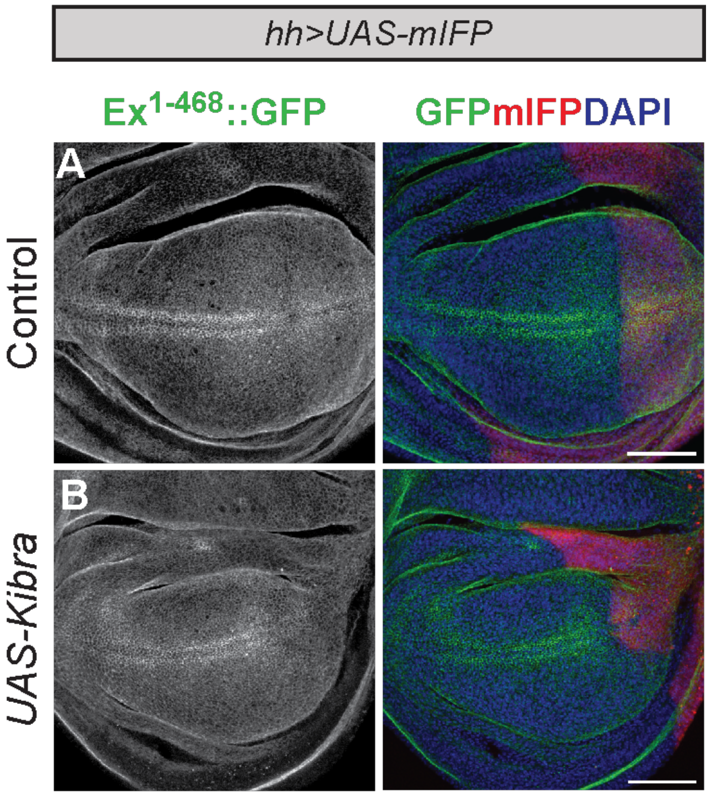

Rebuttal Figure 1: In vivo assessment of the role of Kibra in the regulation of the Ex protein stability reporter. Confocal micrographs depict XY sections of third instar wing imaginal discs expressing *ubi-Ex$^{1\text{-}468}$::GFP* (green) and *UAS-mIFP* (red) alone (control, A) or in combination with UAS-Kibra (B) under the control of the *hh-Gal4* driver (*hh-Gal4* drives transgene expression in the posterior compartment of the developing wing). Nuclei were stained with DAPI (blue). Scale bars correspond to 50µm.

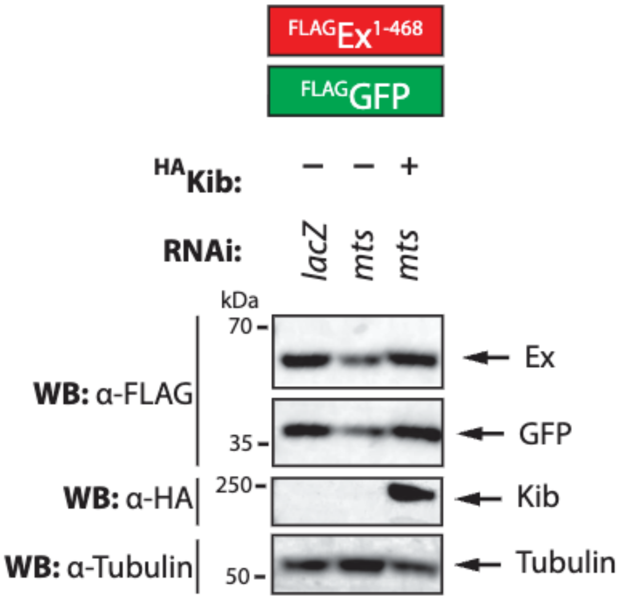

Rebuttal Figure 2: In vitro assessment of the role of Mts in Kibra-mediated stabilisation of Ex protein levels. *Drosophila* S2 cells were transfected with the indicated plasmids 24h after treatment with the indicated RNAi for downregulation of gene expression. 48h after transfection, cells were lysed and processed for Western blot analysis with the indicated antibodies. Note that depletion of *mts* resulted in an intermediate phenotype compared to expression of Kibra alone, which leads to Ex stabilisation. FLAG GFP and tubulin were used as transfection and protein loading controls, respectively.

Reviewer #1 minor comments

1: The Introduction gives a quite comprehensive review of known interactions between STRIPAK, Expanded and Hippo pathway components. However, it is hard to keep track of all the components and interactions if you are not deeply into the field. To improve accessibility, I would suggest a summary diagram of the key interactions (currently the manuscript has no introductory figures at all!) and if possible the authors might consider whether there are details they could leave out or which could just be mentioned as necessary in the results sections.

We thank the reviewer for this suggestion. We agree that the components involved in the molecular mechanism described are very numerous and this can be confusing for a more general audience. We have now included a schematic to illustrate the different regulatory steps in Figure 1A.

2: Could the authors show a shorter exposure of the Ex blot in Figure 1A, in order to better visualize the loss of band shift?

We now include a shorter exposure of the Ex blot in Figure 1B to highlight the fact that Mts expression leads to increased levels of the non-phosphorylated version of Ex.

3: Line 307 '(Fig. 1B,D,G,I)' the call-out to Fig.1I appears to be in strike-through font, presumably because 1I shouldn't be cited here? It also looks like Fig.1I is wrongly cited on line 342 as that sentence only describes action of L168A in wing discs. I think a sentence describing the experiment in Fig.1I is missing?

We apologise for the oversight. The figure citations have been corrected in the revised manuscript.

4: Line 355 ambiguous, should this read low expression of Crb in S2 cells?

We thank the reviewer for highlighting this point. We have rephrased this to clarify that given that PP2A regulated Ex protein levels in a catalytic-site dependent manner in the experiments shown in Figure 2, Ex appears to be phosphorylated even in the absence of ectopic expression of Crb^{intra}.

5: Line 369 reads 'PP2A was able to stabilize full-length Ex', Mts-WT would be more precise.

This has now been altered to address the reviewer's concern.

6: The blot in panel 2O is mislabeled Ex1-468, I think this should be Ex1-450.

We thank the reviewer for spotting this error and it has been corrected.

7: The nomenclature of 'Mts-WT' for their own transgene and 'Mts-BL' for the Bloomington transgene. is confusing, as both are, I believe, wild type. Maybe leave this detail for the M&M, at least if the authors believe there is no difference in behavior.

We apologise for the confusion. Indeed, the transgene we refer to as Mts^{BL} is a wild-type version of Mts. We have clarified this in the main text and in the Materials and Methods to highlight that we have tested two independent constructs.

8: Figure S6 appears to be missing from the uploaded version.

We apologise for this oversight as this was mistakenly omitted from the submission. Figure S6 has now been included in the revised manuscript files.

9: Lines 480-481: 'Using co-IP analyses, we observed that Mts interacts with Ex, both in the presence and absence of Crbintra.' No figure call-out is given for this statement, and I can't see the data anywhere, but from the figure legends it seems to be in the missing Fig.S6? And everything that follows in this paragraph should have call-outs for Fig.4K?

This has now been rectified. Figure S6 is now present in the revised version and the correct figure call-outs have been included in this section.

10: Lines 503-504: 'we found that Kib associated with Mts (Fig. 5C)' - Fig.5B?

This has now been corrected.

11: Lines 504-505: 'no interaction was observed between Mts and Mer (Fig.5B)' - Fig.5C?

This figure call-out has been corrected.

12: In Figure 6G, authors note that 'the mean diap1GFP4.3 levels of MtsWT+Crb-Intra were lower than those of Crb-Intra, this difference was not statistically significant when all genotypes were included in the comparisons, but only when the Control, crbintra and mtsWT+crbintra conditions were considered.' It might be useful to have a table showing the actual P values of all the comparisons (or maybe better still just put actual P values on the graphs?). Sometimes an arbitrary cut-off of 0.05 for significant can be misleading.

We have now included p-value for all the comparisons conducted for all the graphs as supplementary. For Fig.6, we have included the p-values for comparison between Control, Crb^{intra} and Mts^{WT}+Crb^{intra} in the graph, since we explicitly mention these conditions in the text.

Reviewer #2 comments

Significance

The studies strengths include biochemical and in vivo validation of the effect of PP2A and its various regulatory subunits on Ex phosphorylation and stabilization. The study very methodically parses out the context in which PP2A is stabilizing Ex (i.e., both in the context of Crb stimuli and independently, and it does so independently of the STRIPAK complex). As noted previously, recapitulating the major results in clones using genomic alleles would strengthen the biological relevance. The study advances our understanding of mechanisms tightly controlling downstream transcriptional outputs of the Hpo pathway via regulating Ex protein stability/turnover. Though the primary audience may be those well-versed in the Hpo field and Drosophila genetics, the implications for the research are broad.

Evidence, reproducibility and clarity

The authors show that the protein phosphatase PP2A antagonizes Crb-mediated phosphorylation and subsequent degradation of Expanded in vivo. Using Drosophila imaginal wing discs and the GAL4-UAS system, the authors provide evidence that the PP2A holoenzyme dephosphorylates Ex, stabilizing its protein levels, in a manner independent of the STRIPAK complex and identifies Wrd as a key regulatory subunit of PP2A in this process. Importantly, the study also shows that PP2A stabilizes Ex protein levels independent of Crb-driven phosphorylation and that, via this stabilization, PP2A activates Hpo pathway signaling to repress transcriptional targets of Yki.

We thank the reviewer for their assessment of the significance and clarity of our report.

Reviewer #2 major comments

1: Overall, the study is strong, and the conclusions are supported by the data. The data does largely lean on overexpression models in the wing disc and it would strengthen the biological relevance to include genomic alleles (i.e., do Ex-GFP levels go down in PP2A/mts mutant clones?). Materials and methods are thoroughly presented, and statistical analyses are adequate. OPTIONAL: While not necessarily required for publication, note that full in vivo confirmation would require altering the PP2A target sites in Ex by generating phospho-deficient and phospho-mimetic versions and seeing if they match the model. This would push the conclusions to the highest degree of confidence and rigor.

We thank the reviewer for their assessment of our study. We agree with the reviewer that our conclusions would be enhanced by the analysis of PP2A/mts mutant clones and, indeed, this was something we attempted (Rebuttal Figure 3). We generated *mts* mutant clones but these have a very strong phenotype and, invariably, virtually no clones are retrieved, most likely because these cells undergo cell death given that Mts has multiple essential functions in cells. We occasionally recovered single cell clones, but it was too challenging to analyse the Ex::GFP signal in these conditions. We also generated fly stocks to perform MARCM analysis of *mts* mutant clones combined with the Ex::GFP reporter and UAS-Mts transgenes but, again, the loss of Mts resulted in clones that were too small to analyse. We have preliminary evidence that suggests that our transgenes recapitulate our observations as we recovered very small clones with the UAS-Mts^{WT} with scattered single cells found in wing discs.

Regarding the point on the phospho-deficient or phospho-mimetic versions, again we agree with the reviewer that this would be very valuable information. In agreement with our previous publications, we provide evidence that S453 is an important aa residue for the control of Ex protein levels as this is essential for Slimb recognition and degradation. However, as is evident in Figure 2, mutation of S453 alone is insufficient to abrogate the effect of Mts in the absence of ectopic Crb expression and this suggests that other phosphorylation sites may also be important. The main problem with generating mutants that affect Ex phosphorylation is that the N-terminus of Ex and specifically the region that surrounds the Slimb degron is rich in Ser and Thr residues. Hence, we instead opted for the analysis of Ex truncations. Based on our data, we can suggest that, in the absence of ectopic Crb, Mts regulates Ex protein levels at least partly independently of the known Slimb degron as the Ex 1-450 truncation is stabilised by Mts in a catalytically-dependent manner. This suggests that other, hitherto unidentified phosphorylation sites may also be important for this regulation but we believe their identification is beyond the scope of this report as it may be very challenging to pinpoint them.

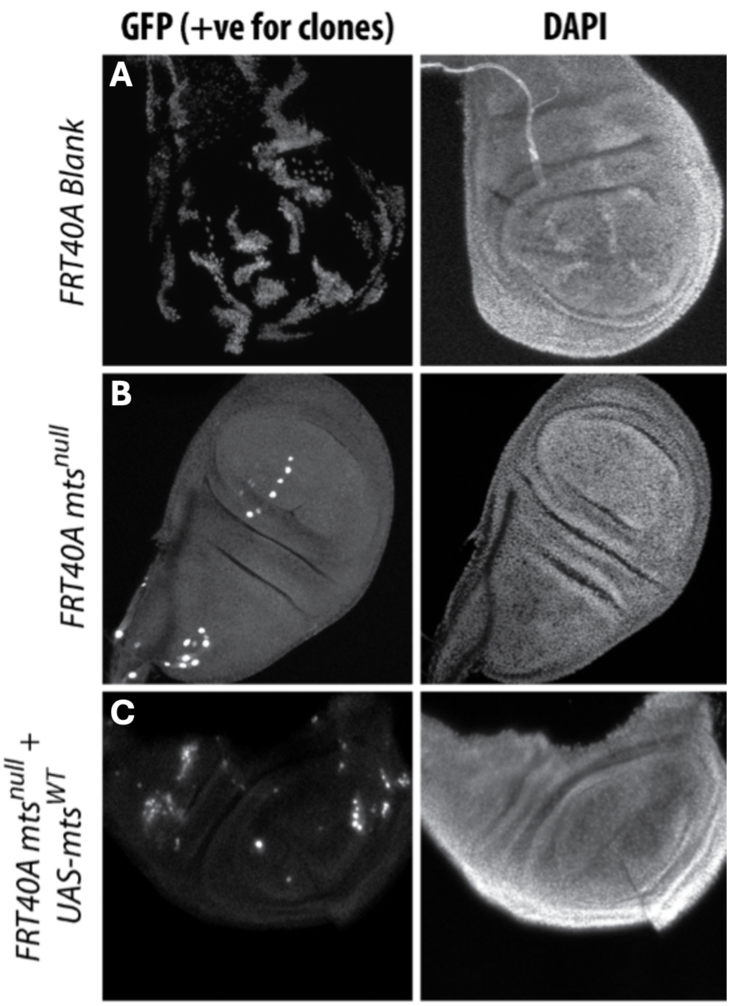

Rebuttal Figure 3: MARCM clone analysis of loss-of-function of mts. XY confocal micrograph sections (A-C) of wing imaginal discs with FRT40A blank MARCM clones (A), mts^{null} mutant clones (B) and mts^{null} mutant clones expressing UAS-mts^{WT} (C). Clones marked by the presence of GFP (left panel); DAPI (right panel).

Reviewer #2 minor comments

1: Text and figures are clear and accurate. It may be helpful to include a modified version of the Mts mutants table in SF1 in a main figure for easier reference but is not necessary.

This has been altered and the information regarding the Mts mutants is now shown in Figure 1C.

Reviewer #3 comments

Significance

A vast amount of data is presented in both in vivo and in vitro settings. The study uses biochemical and genetic approaches and combines them aptly. I think the findings showing multiple and various effects on PP2A on the same pathway would be of higher interest to the PP2A enthusiasts than the Hippo researchers.

Evidence, reproducibility and clarity

The authors hypothesized that Crb mediated Ex phosphorylation and degradation, that they previously established, should be countered and set on to identify the phosphatase involved. Surprisingly, they find that Mts, the catalytic subunit of PP2A, counters the effect of ectopically expressed intracellular domain of Crb on Ex stability. This was surprising because PP2A and the STRIPAK complex was shown to counter Hippo activity previously, suggesting that PP2A would inject both positive and negative inputs into Hpo activity. The title reflects this finding.

Overall, the experiments are well controlled and are of high quality. I especially appreciate the effort to show results of parallel experiments both in S2 cells and in vivo in wing discs.

We thank the reviewer for their assessment of the significance and clarity of our report. We are particularly pleased by their assessment of the importance of conducting parallel experiments in S2 cells and *in vivo* to strengthen our arguments.

Reviewer #3 comments

1: The manuscript convincingly demonstrates that Mts expression stabilizes Ex1-468::GFP in the presence or absence of ectopic Crb-intra. This effect is mainly mediated by the Wrd adaptor subunit, and requires the catalytic activity of Mts. However, results shown in Fig4K highlights the Tws adaptor as the main one that binds to and stabilizes Ex in S2 cells, in the presence or absence of Crb-intra expression. This is slightly at odds with Wrd-RNAi experiments nicely reversing the effects of Crb-intra expression.

As discussed in the manuscript, we believe that Tws potentially represents a constitutive mechanism for regulating Ex protein levels that would occur regardless of the Crb status. This would be consistent with the fact that Tws interacts with Ex independently of the presence of ectopic Crb and it suggests that the molecular mechanism(s) used by Tws are likely to be distinct to those used by Wrd. In contrast, we believe Wrd is primarily involved in the regulation of Ex protein levels in the context of Crb function. Since Ex has phosphosites in addition to the Slmb phosphodegron that are regulated in the absence of ectopic Crb^{intra}, it is possible that Tws regulates Ex stability via these non-Slmb phosphodegron. We hypothesise that Crb interaction with Ex triggers its degradation but, at the same time, promotes the Ex:Wrd interaction that would result in decreased Ex phosphorylation and decreased degradation.

2: The manuscript is not easy to read given the vast amount of data using many different constructs, but there is little the authors can do about it as the story is complex and layered.

We have endeavoured to simplify as much as possible the manuscript but, as the reviewer rightly highlights, it is challenging to maintain the message when analysing so many different proteins.

3: The argument that the effects of Mts are independent of the STRIPAK complex is less convincing. This conclusion is based on Mts-L186A mutant which should not bind Cka which is the PP2A adaptor subunit found in the STRIPAK complex. Fig S3F and G show that Cka binding to Mts is reduced by half when Mts-L186A mutant is expressed in lieu wt Mts. Consistent with this in Fig1F rescue of Ex degradation by Mts-L186A is half as effective as the rescue seen in 1F by the wt Mts. Towards the same argument, data shown on S3A-D is deemed inconclusive based on quantification in S3E which does not reflect the clear reduction in Ex that is seen in S3B. Hence FigS3 is in favour of Cka4 being involved in the rescue effect.

We acknowledge the reviewer's comment and concern regarding our interpretation of the role of the STRIPAK complex in this process. As mentioned in the response to reviewer #1, the fact that the Mts mutants generated do not totally affect the binding of Mts to the regulatory subunits as expected from structural studies complicates the interpretation of the data. To address the concerns raised by both reviewers, we performed experiments where we combined the Mts^{L186A} mutation with RNAi-mediated depletion of *wrd* (Fig. 3K,L,O).. Our data show that depletion of *wrd* abrogates the effect of Mts^{L186A}, strongly suggesting that any effect of Mts^{L186A} on the regulation of Ex protein levels is indeed due to its ability to bind Wrd and not due to any residual binding to Cka. If the Mts^{L186A}:Cka complex had an essential function, we would expect that it would still be able to compensate for *wrd RNAi* and this is clearly not the case. Therefore, this strongly suggests that the STRIPAK is not essential for the effect of PP2A in this instance.

4: In Figures 5A and 3A, Crb-intra expression does not destabilize Ex1-468::GFP, why is that?

This is due to the expression levels of Crb^{intra} in this particular biological repeat of the experiment. However, we can clearly see that Mts can dephosphorylate and further stabilise Ex in these blots.

5: The authors connect effects on Ex stability to the influence on Hippo pathway activity in Fig 6, which is a very nice touch.

We thank the reviewer for the assessment of our experimental approaches linking Ex regulation and the role of PP2A to Hippo pathway readouts.

6: Finally, I wonder whether the dual effect of PP2A on Hippo activity (inhibiting Hippo and stabilizing Ex) could be a single effect. I am guessing the Ex1-468::GFP construct, having its own regulatory elements, would act independently of the transcriptional activity of Hippo. However, I was not able to find this demonstrated in the literature. Can the authors show that? For example, make hpo or wts mutant clones in the presence of the Ex1-468::GFP construct.

Otherwise, an alternative explanation could be that PP2A, with its various adaptor subunits, counters Hippo activity which translates into higher levels of expanded transcription and Ex protein production. It was also demonstrated that there are higher levels of Crb in hippo mutants likely due to the expansion of the apical domain. This would be consistent with the stabilized Crb-intra seen in Figures 1A&3A upon Mts expression. Stabilization of Crb upon Mts expression (not commented on in the manuscript) is very interesting as extra Crb should further push the balance towards Ex degradation but Mts seems to be able to reverse the effect. I agree that this alternative explanation may be far-fetched, yet it is also easily tested, and would greatly simplify the model put forward.

We thank the reviewer for the considerations regarding the regulatory mechanisms involved. The reviewer is correct in their interpretation of the features of the Ex::GFP reporter. The reporter is under the control of the *ubiquitin 63E* promoter as opposed to the endogenous *ex* promoter. As such, we do not envisage that the transcription of the reporter is regulated by Yki and we have confirmed this *in vivo* (shown in Fig. S2C-F). Indeed, a similar method of decoupling potential transcriptional and post-transcriptional effects of Hippo signalling has been successfully used in studies that have focused on other Hippo pathway components, such as Kibra (Tokamov et al. 2021, PMID: 33555257) and Salvador (Aerne et al. 2015, PMID: 26125558).The reviewer also suggests that assessing the effect of *hpo* and *wts* mutant clones on the *ubi-Ex1-468::GFP* reporter would be informative. However, we believe that this would lead to results that will be difficult to interpret. Although *hpo* and *wts* mutant clones would result in higher Yki activity, they would also remove Hpo or Wts function, and these proteins may be involved in the molecular mechanisms that regulate Ex protein stability and, in the case of Wts, that is indeed the case (Zhang et al. 2015, PMID:25522691). Finally, the reviewer also suggests that Mts may be involved in regulating Crb^{intra} levels. We have addressed this point and, at least in the conditions tested, we observed no effect of Mts on the stability of Crb^{intra} in S2 cells (shown in Fig. S1A).

7: Finally, if indeed various PP2A complexes, depending on the adaptor subunits they contain, have a range of effects on Ex stability and Hippo pathway activity, this brings in the question of what regulates the availability of various adaptor subunits and the PP2A complexes they form? The question is outside the scope of the manuscript but it is worth discussing.
This is indeed one of the outstanding questions that is raised by our report. Given that we have identified an antagonistic role for PP2A complexes in the regulation of Hippo signalling, one would predict that the more downstream events in the pathway would be dramatically influenced by which PP2A complex is more prominent. As our results have shown (Fig. 6), we can promote one of the functions of PP2A by ectopically expressing specific Mts mutants that affect the ability of PP2A to participate in particular PP2A complexes (e.g. $PP2A^{STRIPAK}$ vs $PP2A^{Wrd}$). Ultimately, the complexes identified contain different regulatory subunits and, therefore, the regulation is likely to happen at this level. We agree that it is of particular interest to reveal these additional layers of regulation, but we believe that this falls outside the scope of this report, particularly as some of the experiments required to gain further insight would most likely require the generation of new tools (e.g. tagged PP2A regulatory subunits for precise subcellular localisation) and optimisation of technical approaches (e.g. live imaging of developing wing discs). As suggested by the reviewer, we have added this to our discussion section (Lines 649-664).

Overall reviewer comments
1: is the effect really independent of STRIPAK?
2: do the effects seen on ectopic Ex1-468 apply to endogenous Ex?

A relatively simple experiment could possibly address both issues. If the model is correct and PP2A can target both Hippo and Ex using different adaptor proteins, then we would expect modulating the levels of Tws and Wrd adaptors to influence Ex stability, but not Hpo phosphorylation. Could the authors test this hypothesis in vivo, looking at the endogenous proteins?
This is an interesting point to test. However, in our hands, we were unable to see any noticeable differences in p-Hpo levels when Crbintra was expressed (Rebuttal Figure 4). Hence, it would be difficult to conduct the experiments suggested by the reviewers.

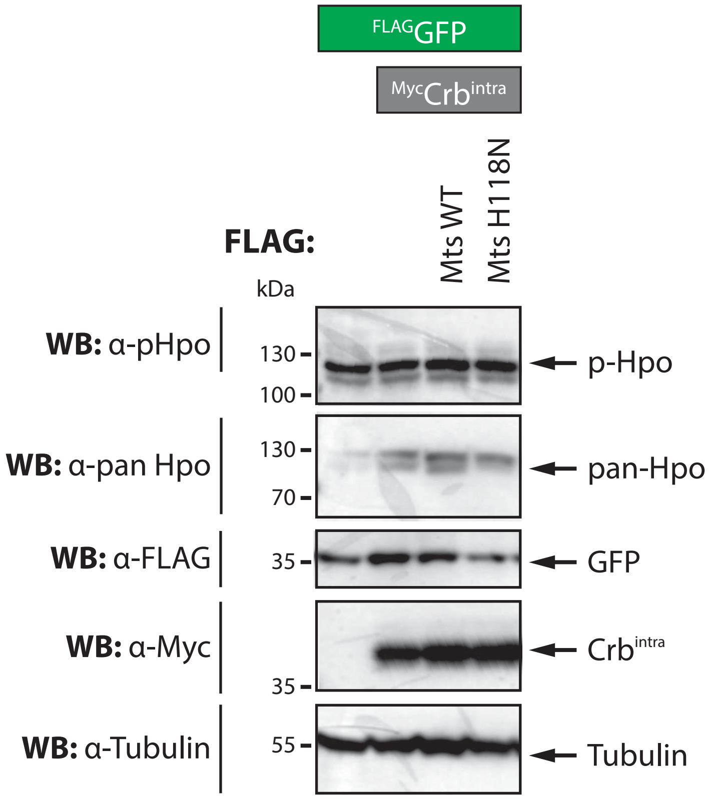

Rebuttal Figure 4: Effect of Crb and PP2A on p-Hpo levels. S2 cells were transfected with the indicated constructs and analysed 48h after transfection via immunoblot with the indicated antibodies.

1st Revision - Editorial Decision 15th Jun 2026

Dear Dr Ribeiro,

Thank you again for submitting of your amended manuscript (EMBOJ-2025-120833R | [RC-2024-02819]) to The EMBO Journal. Please accept my sincere apologies for the unusual protraction with the reassessment of your work due to delayed expert input and detailed discussion in the editorial team. Your revised study was sent back to the referees for their scientific reassessment, and we have received detailed re-reports from two of them, which I attach below. Please note that while referee #3 was not able to re-evaluate your amended study at this time, we have editorially assessed your response to the issues raised and found them to be addressed satisfactorily. As you will see, the other referees state that the work has been substantially enhanced by the revisions and they are now in favour of publication, pending minor revision.

Thus, we are pleased to inform you that your manuscript has been accepted in principle for publication in The EMBO Journal.

Please carefully consider the remaining minor points by referee #2 by adding complementary data or adjusting the claims and discussion of the results in the manuscript text by introducing caveats where appropriate.

Also, we now need you to take care of a number of minor issues related to formatting and data annotation as detailed below.

Please let us know any time if there are questions related.

As you might have seen on our web page, every paper at the EMBO Journal now includes a 'Synopsis', displayed on the html and freely accessible to all readers. The synopsis includes a 'model' figure as well as 2-5 one-short-sentence bullet points that summarize the article. I would appreciate if you could provide this figure and the bullet points.

Please submit a revised version of the manuscript using the link enclosed below.

Thank you again for giving us the chance to consider your manuscript for The EMBO Journal.

I look forward to receiving your final revised version of the manuscript for swift acceptance and online publication.

Kind regards,

Daniel Klimmeck

Daniel Klimmeck PhD
Senior Editor
The EMBO Journal

******

Formatting changes required for the revised version of the manuscript:

>> Please add a 'Disclosure and Competing Interests Statement' after the Acknowledgements.

>> Please correct the order and headings of the sections in the manuscript text to: Abstract / Introduction / Results / Discussion / Methods / Data Availability / Acknowledgements / Disclosure and Competing Interests Statement / References / Figure Legends / Expanded View Figure Legends.

>> Figures: rename the suppl. figures to "Figure EV1" - "Figure EV6"

>> Figure callouts: Please ensure that the figures and panels are called out in sequential order. Currently, a callout is missing for Fig 4D.

>> References: please adjust the reference format to EMBO Journal format, 10 authors et al.

>> Please provide source data for the study as to the separate request e-mail. Source data should be uploaded as one (zipped) file per figure. Currentyl, source data for Fig. 4A-H are missing.

>> Remove the 'data not shown' statement on p.18, line 331 ("which were consistently observed at 25{degree sign}C...."), or add respective information.

>> Data availability section: please change the statement to 'No large-scale data amenable to data repository deposition were generated in this study.' . Adjust the Author Checklist accordingly.

>> Consider additional changes and comments from our production team as indicated below:
- Figure legends:

1.. Please note that the exact p values are not provided in the legends of figures 1I, 2E, J; 3B, M-O; 4I, J; 4I, J; S2F, J; S3 A, B, H; S5 D, E
2. Please note that the exact p values are not provided in the legends of figures 1I, 2E, J; 3B, M-O; 4I, J; 4I, J; S2F, J; S3 A, B, H; S5 D, E

Further information is available in our Guide For Authors: https://link.springer.com/journal/44318/submission-guidelines

----

Referee #1:

The paper nicely shows that PP2A antagonizes Crb-dependent and Crb-independent phosphorylation and degradation of Expanded (Ex), in cell culture and in wing discs. The authors focus on the Mts catalytic subunit of PP2A, but also demonstrate the involvement of the Wrd and Tws B regulatory subunits. They also show via use of transcriptional reporters that PP2A directly affects Hpo signaling in vivo. Finally, they show a potential role for Merlin and Kibra in regulating Ex levels, and that Kib binds to Mts and Wrd. The experiments are on the whole well executed and quantified.

The authors have addressed all my comments, and I am now happy for the manuscript to be published as is.

Referee #2:

The paper addresses a gap in knowledge in the Drosophila Hippo pathway relating to Crumbs-induced phosphorylation of the Expanded tumor suppressor protein, which triggers Expanded degradation. The authors set out to identify the phosphatase(s) that opposes this mechanism. Surprisingly, they determined that a PPase2A (PP2A) subunit, Microtubule star (Mts), that is required to dephosphorylate and inactivate Hippo kinase through a role in the STRIPAK complex is also required to dephosphorylate and stabilize Expanded. Through the use of point mutant versions and RNAi approaches, the authors are able to demonstrate the Mts engages different substrate adaptors to execute these two opposing roles; the Wrd subunit is required in

vivo to stabilize Expanded, while the Cka subunit, which Mts binds to inactivate Hippo, is dispensable. The data overall and well executed and well presented.

Points:
The paper is strong on S2 co-IPs and in vivo genetics, but it sidesteps molecular analyses of Expanded phosphorylation, which is at the core of the story. Expanded p-state is especially interesting given their evidence that Mts-Wrd regulation of Expanded is independent of the known phosphorylation site in the Slmb degron. The authors logically infer logically that there must be additional p-sites in Expanded that play importrant regulatory roles. But there is no attempt to test this hypothesis, even with a simple +/-PPase mobility shift in SDS-PAGE or by a more direct test using phos-tag gels. Having a better idea of the number of p-sites in Expanded might help resolve elements of the model if it turns out that a subset are Crb independent or selectively regulated by Tws or Wrd.

The interactions between Kibra or Merlin and PPase subunits in S2 cells are exciting (Fig 5) but they need to be validated in vivo, as in prior figures. This would be a nice addition, but these data don't bear directly on the core findings and are of lower priority.

line 532: the references for Crb[intra} regulating ex-Z and diap-Z are incorrect. The relevant papers were published in 2010.

***
Rev_Com_number: RC-2024-02819
New_manu_number: EMBOJ-2025-120833R
Corr_author: Ribeiro
Title: A Dual Role for the PP2A Phosphatase in Hippo Signalling Regulation

The authors addressed the remaining editorial issues.

Dear Dr Ribeiro,

Thank you for submitting the revised version of your manuscript. I have now evaluated your amended manuscript and concluded that the remaining minor concerns have been sufficiently addressed.

I am thus pleased to inform you that your manuscript has been accepted for publication in the EMBO Journal.

Your manuscript will be processed for publication by EMBO Press. It will be copy edited and you will receive page proofs prior to publication. Please note that you will be contacted by Springer Nature Author Services to complete licensing and payment information.

Please note that it is The EMBO Journal policy for the transcript of the editorial process (containing referee reports and your response letter) to be published as an online supplement to each paper. If you do NOT want this, you will need to inform the Editorial Office via email immediately. More information is available here: https://www.embopress.org/transparent-process#Review_Process

You may qualify for financial assistance for your publication charges - either via a Springer Nature fully open access agreement or an EMBO initiative. Check your eligibility: https://www.embopress.org/page/journal/14602075/authorguide#chargesguide

Should you be planning a Press Release on your article, please notify the production team via this form in order to coordinate publication and release dates: https://forms.cloud.microsoft/e/eH4A8JVP6g

If you have any questions, please do not hesitate to contact the Editorial Office. Thank you for your contribution to The EMBO Journal.

Thank you again for this contribution to The EMBO Journal and congratulations on a successful publication! Please consider us again in the future for your most exciting work.

Best regards,

Daniel Klimmeck

Daniel Klimmeck, PhD
Senior Editor
The EMBO Journal
EMBO
Postfach 1022-40
Meyerhofstrasse 1
D-69117 Heidelberg
contact@embojournal.org

*****
Please note that it is The EMBO Journal policy for the transcript of the editorial process (containing referee reports and your response letters) to be published as an online supplement to each paper. If you should prefer removal of any referee-only figures included in the point-by-point response(s), e.g. because they may still be used for future publication or because they have been reproduced from published work by others, please do let us know immediately via response email.

More information is available here: https://link.springer.com/partners/embo-press/editorial-policies#Peer%20review

** Click here to be directed to your login page: https://emboj.msubmit.net

EMBO Press Author Checklist

Corresponding Author Name: Paulo Ribeiro, Aashika Sekar
Journal Submitted to: EMBO Journal
Manuscript Number: EMBOJ-2025-120833-T | [RC-2024-02819]

USEFUL LINKS FOR COMPLETING THIS FORM
The EMBO Journal - Author Guidelines
EMBO Reports - Author Guidelines
Molecular Systems Biology - Author Guidelines
EMBO Molecular Medicine - Author Guidelines

Reporting Checklist for Life Science Articles (updated January
This checklist is adapted from Materials Design Analysis Reporting (MDAR) Checklist for Authors. MDAR establishes a minimum set of requirements in transparent reporting in the life sciences (see Statement of Task: 10.31222/osf.io/9sm4x). Please follow the journal's guidelines in preparing your
Please note that a copy of this checklist will be published alongside your article.

Abridged guidelines for figures
1. Data
The data shown in figures should satisfy the following conditions:
- the data were obtained and processed according to the field's best practice and are presented to reflect the results of the experiments in an accurate and unbiased manner.
- ideally, figure panels should include only measurements that are directly comparable to each other and obtained with the same assay.
- plots include clearly labeled error bars for independent experiments and sample sizes. Unless justified, error bars should not be shown for technical
- if n<5, the individual data points from each experiment should be plotted. Any statistical test employed should be justified.
- Source Data should be included to report the data underlying figures according to the guidelines set out in the authorship guidelines on Data

2. Captions
Each figure caption should contain the following information, for each panel where they are relevant:
- a specification of the experimental system investigated (eg cell line, species name).
- the assay(s) and method(s) used to carry out the reported observations and measurements.
- an explicit mention of the biological and chemical entity(ies) that are being measured.
- an explicit mention of the biological and chemical entity(ies) that are altered/varied/perturbed in a controlled manner.
- the exact sample size (n) for each experimental group/condition, given as a number, not a range;
- a description of the sample collection allowing the reader to understand whether the samples represent technical or biological replicates (including how many animals, litters, cultures, etc.).
- a statement of how many times the experiment shown was independently replicated in the laboratory.
- definitions of statistical methods and measures:
  - common tests, such as t-test (please specify whether paired vs. unpaired), simple χ2 tests, Wilcoxon and Mann-Whitney tests, can be unambiguously identified by name only, but more complex techniques should be described in the methods section;
  - are tests one-sided or two-sided?
  - are there adjustments for multiple comparisons?
  - exact statistical test results, e.g., P values = x but not P values < x;
  - definition of 'center values' as median or average;
  - definition of error bars as s.d. or s.e.m.

Please complete ALL of the questions below.
Select "Not Applicable" only when the requested information is not relevant for your study.

Materials

Newly Created Materials	Information included in the manuscript?	In which section is the information available? (Reagents and Tools Table, Materials and Methods, Figures, Data Availability Section)
New materials and reagents need to be available; do any restrictions apply?	Yes	Materials and Methods; No restriction

Antibodies	Information included in the manuscript?	In which section is the information available? (Reagents and Tools Table, Materials and Methods, Figures, Data Availability Section)
For **antibodies** provide the following information:<br>- Commercial antibodies: RRID (if possible) or supplier name, catalogue number and or/clone number<br>- Non-commercial: RRID or citation	Yes	Materials and Methods

DNA and RNA sequences	Information included in the manuscript?	In which section is the information available? (Reagents and Tools Table, Materials and Methods, Figures, Data Availability Section)
**Short novel DNA or RNA including primers, probes**: provide the sequences.	Yes	Materials and Methods

Cell materials	Information included in the manuscript?	In which section is the information available? (Reagents and Tools Table, Materials and Methods, Figures, Data Availability Section)
**Cell lines:** Provide species information, strain. Provide accession number in repository **OR** supplier name, catalog number, clone number, and/**OR** RRID.	Yes	Materials and Methods
**Primary cultures:** Provide species, strain, sex of origin, genetic modification status.	Not Applicable	
Report if the cell lines were recently **authenticated** (e.g., by STR profiling) and tested for mycoplasma contamination.	Not Applicable	

Experimental animals	Information included in the manuscript?	In which section is the information available? (Reagents and Tools Table, Materials and Methods, Figures, Data Availability Section)
**Laboratory animals or Model organisms:** Provide species, strain, sex, age, genetic modification status. Provide accession number in repository **OR** supplier name, catalog number, clone number, **OR** RRID.	Yes	Materials and Methods
**Animal observed in or captured from the field:** Provide species, sex, and age where possible.	Not Applicable	
Please detail **housing and husbandry conditions**.	Not Applicable	

Plants and microbes	Information included in the manuscript?	In which section is the information available? (Reagents and Tools Table, Materials and Methods, Figures, Data Availability Section)
**Plants:** provide species and strain, ecotype and cultivar where relevant, unique accession number if available, and source (including location for collected wild specimens).	Not Applicable	
**Microbes:** provide species and strain, unique accession number if available, and source.	Not Applicable	

Human research participants	Information included in the manuscript?	In which section is the information available? (Reagents and Tools Table, Materials and Methods, Figures, Data Availability Section)
If collected and within the bounds of privacy constraints report on age, sex and gender or ethnicity for all study participants.	Not Applicable	

Core facilities	Information included in the manuscript?	In which section is the information available? (Reagents and Tools Table, Materials and Methods, Figures, Data Availability Section)
If your work benefited from core facilities, was their service mentioned in the acknowledgments section?	Yes	Acknowledgements

Design

Study protocol	Information included in the manuscript?	In which section is the information available? (Reagents and Tools Table, Materials and Methods, Figures, Data Availability Section)
If study protocol has been **pre-registered, provide DOI in the manuscript**. For clinical trials, provide the trial registration number **OR** cite DOI.	Not Applicable	
Report the **clinical trial registration number** (at ClinicalTrials.gov or equivalent), where applicable.	Not Applicable	

Laboratory protocol	Information included in the manuscript?	In which section is the information available? (Reagents and Tools Table, Materials and Methods, Figures, Data Availability Section)
Provide DOI OR other citation details if **external detailed step-by-step protocols** are available.	Not Applicable	

Experimental study design and statistics	Information included in the manuscript?	In which section is the information available? (Reagents and Tools Table, Materials and Methods, Figures, Data Availability Section)
Include a statement about **sample size** estimate even if no statistical methods were used.	Yes	Figure Legends where applicable
Were any steps taken to minimize the effects of subjective bias when allocating animals/samples to treatment (e.g. **randomization procedure**)? If yes, have they been described?	Not Applicable	
Include a statement about **blinding** even if no blinding was done.	Not Applicable	
Describe **inclusion/exclusion criteria** if samples or animals were excluded from the analysis. Were the criteria pre-established? If sample or data points were omitted from analysis, report if this was due to attrition or intentional exclusion and provide justification.	Not Applicable	
For every figure, are **statistical tests** justified as appropriate? Do the data meet the assumptions of the tests (e.g., normal distribution)? Describe any methods used to assess it. Is there an estimate of variation within each group of data? Is the variance similar between the groups that are being statistically compared?	Yes	Materials and Methods, Figure Lengends where applicable

Sample definition and in-laboratory replication	Information included in the manuscript?	In which section is the information available? (Reagents and Tools Table, Materials and Methods, Figures, Data Availability Section)
In the figure legends: state number of times the experiment was **replicated** in laboratory.	Yes	Figure lengends
In the figure legends: define whether data describe **technical or biological replicates**.	Yes	Figure lengends

Ethics

Ethics	Information included in the manuscript?	In which section is the information available? (Reagents and Tools Table, Materials and Methods, Figures, Data Availability Section)
Studies involving **human participants**: State details of **authority granting ethics approval** (IRB or equivalent committee(s), provide reference number for approval.	Not Applicable	
Studies involving **human participants**: Include a statement confirming that **informed consent** was obtained from all subjects and that the experiments conformed to the principles set out in the WMA Declaration of Helsinki and the Department of Health and Human Services Belmont Report.	Not Applicable	
Studies involving **human participants: For** publication of **patient photos**, include a statement confirming that consent to publish was obtained.	Not Applicable	
Studies involving experimental **animals**: State details of **authority granting ethics approval** (IRB or equivalent committee(s), provide reference number for approval. Include a statement of compliance with ethical regulations.	Not Applicable	
Studies involving **specimen and field samples:** State if relevant **permits** obtained, provide details of authority approving study; if none were required, explain why.	Not Applicable	

Dual Use Research of Concern (DURC)	Information included in the manuscript?	In which section is the information available? (Reagents and Tools Table, Materials and Methods, Figures, Data Availability Section)
Could your study fall under dual use research restrictions? Please check biosecurity documents and list of **select agents and toxins** (CDC): https://www.selectagents.gov/sat/list.htm	Not Applicable	
If you used a select agent, is the security level of the lab appropriate and reported in the manuscript?	Not Applicable	
If a study is subject to dual use research of concern regulations, is the name of the **authority granting approval and reference number** for the regulatory approval provided in the manuscript?	Not Applicable	

Reporting

The MDAR framework recommends adoption of discipline-specific guidelines, established and endorsed through community initiatives. Journals have their own policy about requiring specific guidelines and recommendations to complement MDAR.

Adherence to community standards	Information included in the manuscript?	In which section is the information available? (Reagents and Tools Table, Materials and Methods, Figures, Data Availability Section)
State if relevant guidelines or checklists (e.g., **ICMJE, MIBBI, ARRIVE, PRISMA**) have been followed or provided.	Not Applicable	
For **tumor marker prognostic studies,** we recommend that you follow the **REMARK** reporting guidelines (see link list at top right). See author guidelines, under 'Reporting Guidelines'. Please confirm you have followed these guidelines.	Not Applicable	
For **phase II and III randomized controlled trials**, please refer to the **CONSORT** flow diagram (see link list at top right) and submit the CONSORT checklist (see link list at top right) with your submission. See author guidelines, under 'Reporting Guidelines'. Please confirm you have submitted this list.	Not Applicable	

Data Availability

Data availability	Information included in the manuscript?	In which section is the information available? (Reagents and Tools Table, Materials and Methods, Figures, Data Availability Section)
Have **primary datasets** been deposited according to the journal's guidelines (see 'Data Deposition' section) and the respective accession numbers provided in the Data Availability Section?	Yes	Data availability
Were **human clinical and genomic datasets** deposited in a public access-controlled repository in accordance to ethical obligations to the patients and to the applicable consent agreement?	Not Applicable	
Are **computational models** that are central and integral to a study available without restrictions in a machine-readable form? Were the relevant accession numbers or links provided?	Not Applicable	
If publicly available data were reused, provide the respective **data citations in the reference list**.	Not Applicable	
